# Methane emissions from natural gas vehicles in China

**DOI:** 10.1038/s41467-020-18141-0

**Published:** 2020-09-11

**Authors:** Da Pan, Lei Tao, Kang Sun, Levi M. Golston, David J. Miller, Tong Zhu, Yue Qin, Yan Zhang, Denise L. Mauzerall, Mark A. Zondlo

**Affiliations:** 1 Department of Civil and Environmental Engineering, Princeton University, Princeton, NJ 08544 USA; 2 Center for Mid-Infrared Technologies for Health and The Environmental, NSF-ERC, Princeton, NJ 08544 USA; 3 Department of Civil, Structural and Environmental Engineering, University at Buffalo, Buffalo, NY 14260 USA; 4 Research and Education in eNergy, Environment and Water (RENEW) Institute, University at Buffalo, Buffalo, NY 14260 USA; 5 Currently at Environmental Defense Fund, New York, NY 10010 USA; 6 State Key Joint Laboratory for Environmental Simulation and Pollution Control, College of Environmental Sciences and Engineering, Peking University, 100871 Beijing, China; 7 Department of Geography, The Ohio State University, Columbus, OH 43210 USA; 8 Sustainability Institute, The Ohio State University, Columbus, OH 43210 USA; 9 Department of Geography, Environment, and Spatial Sciences, Michigan State University, East Lansing, MI 48824 USA; 10 Woodrow Wilson School of Public and International Affairs, Princeton University, Princeton, NJ 08544 USA

**Keywords:** Atmospheric chemistry, Climate change, Environmental monitoring, Climate-change impacts

## Abstract

Natural gas vehicles (NGVs) have been promoted in China to mitigate air pollution, yet our measurements and analyses show that NGV growth in China may have significant negative impacts on climate change. We conducted real-world vehicle emission measurements in China and found high methane emissions from heavy-duty NGVs (90% higher than current emission limits). These emissions have been ignored in previous emission estimates, leading to biased results. Applying our observations to life-cycle analyses, we found that switching to NGVs from conventional vehicles in China has led to a net increase in greenhouse gas (GHG) emissions since 2000. With scenario analyses, we also show that the next decade will be critical for China to reverse the trend with the upcoming China VI standard for heavy-duty vehicles. Implementing and enforcing the China VI standard is challenging, and the method demonstrated here can provide critical information regarding the fleet-level CH_4_ emissions from NGVs.

## Abstract (web summary)

The methane emissions from natural gas vehicles (NGVs) are unclear. Here the authors report high methane emissions from heavy-duty NGVs, and by using a scenario analysis show that strictly implementing the upcoming China VI standard could reduce GHG emissions by 509 Mt CO_2eq_ for 2020-2030.

## Introduction

**Fig. 1:**
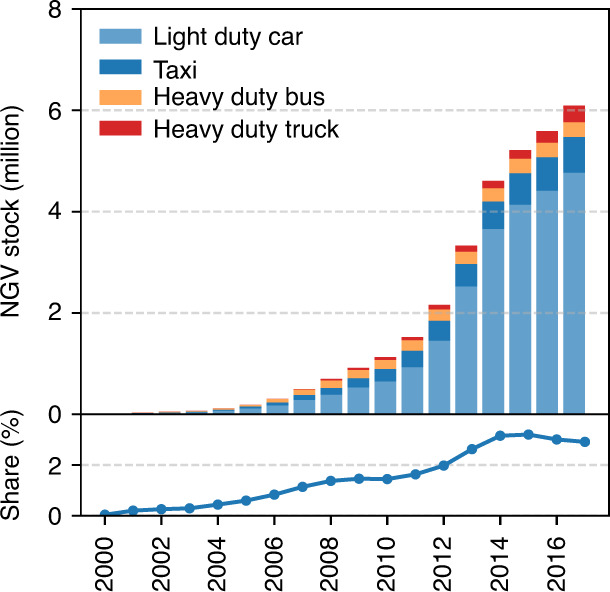
Population of natural gas vehicles (NGVs) and their share in total vehicle stock in China from 2000 to 2017. Supplementary Table S1 lists the number of NGVs in each category. Source data are provided as a Source Data file.

From 2000 to 2017, the population of natural gas vehicles (NGVs) in China increased from 6000 to 6.08 million (Fig. 1 and Supplementary Table 1)^1^. This rapid growth of NGVs in China is primarily driven by environmental considerations and associated economic incentives. Natural gas (NG) is considered to be a clean-burning fuel characterized by relatively low carbon content and low air pollutant emissions^2^, making it less costly for NGVs to meet increasingly stringent PM_2.5_ and NO_*x*_ emission standards in China compared to gasoline and diesel counterparts^3,4^. Currently, the payback time of the additional cost for purchasing a NGV relative to gasoline and diesel counterparts are two to three years for a taxi driver^3^, and around one year for a truck driver in China (see Supplementary Fig. 1 for details). China aims to increase the population of NGVs to 10 million by 2020^5^, and more growth is expected beyond 2020 for heavy-duty applications (buses and trucks) where vehicle electrification remains difficult^6^. In 2017, 6.37 million heavy-duty trucks (all fuel-types combined) accounted for 3.1% of the total vehicles in China and contributed 53 and 60% of the vehicular NO_*x*_ and particulate matter (PM) emissions^7^. To improve the situation, the State Council of the People’s Republic of China set the goal to retire heavy-duty vehicles meeting just China III emission standards and specifically mentioned the goal of promoting the use of heavy-duty NGVs for the first time in 2018^8^. In the first half of 2019, 85,000 heavy-duty NG trucks were sold in China accounting for 13% of the total heavy-duty vehicles sold in the same period and were 27% higher than the annual sales of heavy-duty NG trucks in 2018^9^.

Although NGVs are cost-effective alternatives to achieve the desired NO_*x*_, PM, and CO_2_ emission reductions^4,10,11^, their unintended methane (CH_4_) emissions could compromise their potential climate benefits. CH_4_ is a potent greenhouse gas (GHG) with a global warming potential (GWP) of 28–34 over a 100-year time horizon (84–86 over a 20-year time horizon)^12^. Despite having the largest NGV fleet in the world, emission factors (EFs) of NGVs in China have not been carefully quantified, and CH_4_ emissions from NGVs are not included in the CH_4_ emission inventory for China^13–15^. Previous studies of NGVs mainly focused on CH_4_ emissions from the upstream stages in China, such as extraction, processing, and distribution of NG, using life-cycle analysis (LCA). Emissions related to vehicle operation have been ignored in these life-cycle analyses because of the lack of direct measurements in China^14,16–18^. CH_4_ can be emitted from NGVs as unburnt fuels from tailpipes. CH_4_ is the hydrocarbon most resistant to catalytic oxidation. Therefore, CH_4_ removal rates in the exhaust of NGVs are highly variable and strongly depend on the combination of engine and after-treatment technology^2^. CH_4_ also has been observed in the blowby gas from spark ignited (SI) engines with an open crankcase system and in the vented gas from High-Pressure Direct Injection (HPDI) engines^19^. Occasionally, CH_4_ can be released directly from on-board fuel storage tanks as well due to manual or pressure relief venting^19^. Clark et al.^20^ estimated that vehicle emissions account for about 80% and 40–60% of pump-to-wheels (PTW) and well-to-wheels (WTW) CH_4_ emissions, respectively, based upon observations for NGVs in the US. For WTW GHG emissions from NGVs in China, only Huo et al.^16^ adopted the CH_4_ emission factor (EF) of light-duty NGVs developed for the US. Other life-cycle analyses for China have ignored vehicle CH_4_ emissions from NGVs entirely^14,17,21,22^.

In fact, CH_4_ emissions from NGVs in China may be significantly higher than the results from the US and other regions. In China, about 80% of NGVs are retrofitted from conventional vehicles with engines and after-treatment equipment not designed for NG^23^. In 2018, Hu et al.^23^ reported the overall CH_4_ emission factor of NGVs in China (3.0 ± 0.5% of NG consumed, (mean ± standard error)) is about eight times the emission factor for NGVs given by the IPCC (0.4% of NG consumed). Without distinguishing light- and heavy-duty NGVs (Supplementary Fig. 2), they attributed the high CH_4_ emissions to the retrofitted light-duty NGVs^23^. However, heavy-duty NG buses and trucks produced in China are equipped with lean-burn (LB) engines and oxidation catalysts (OC)^2^. The exhaust temperature of LB engines is usually lower than the ideal temperature for OC (450 °C) to effectively remove CH_4_^2,24^. The mean value of fuel-specific EFs found in previous studies for LB engines with OC is six times higher than that of stoichiometric (SM) engines equipped with a three-way catalyst (TWC, Supplementary Table 2 lists CH_4_ EFs for different technologies)^25^. Finally, although CH_4_ emissions from heavy-duty NGVs are regulated by emission standards in China, the low removal rate at low exhaust temperature may lead to significantly elevated real-world emissions than the certified emission limit. Such discrepancies have been reported widely for NO_*x*_ emissions from heavy-duty vehicles equipped with selective catalytic reduction (SCR) systems, which are also temperature sensitive^10,26^. Because measurements of CH_4_ emissions from NGVs are lacking, however, it is unclear whether such a discrepancy exists for CH_4_ emissions from NGVs in China and to what extent it impacts their GHG emissions.

During the 2014 CAREBEIJING North China Plain field campaign, we deployed a mobile laboratory to quantify CH_4_ EFs of NGVs in China. We updated the well-to-wheels GHG emissions for NGVs with the observed EFs and developed a detailed bottom-up CH_4_ emission inventory of NGVs in China for 2000–2017. Starting July 1st, 2019, heavy-duty NGVs sold in China must be certified for the China VI emission standard for heavy-duty vehicles^27^. We designed three scenarios to assess the potential impacts of the implementation of the new standard on CH_4_ emissions from NGVs. Our results show that CH_4_ emissions from heavy-duty NGVs were high and switching to NGVs from conventional vehicles in China has led to a net increase of 77 Mt CO_2eq_ from 2000–2017. Our scenario analyses demonstrate that strictly implementing the upcoming China VI standard could reduce GHG emissions by 509 Mt CO_2eq_ for 2020–2030.

## Results

### On-road CH_4_ emissions from NG taxis and buses

**Fig. 2:**
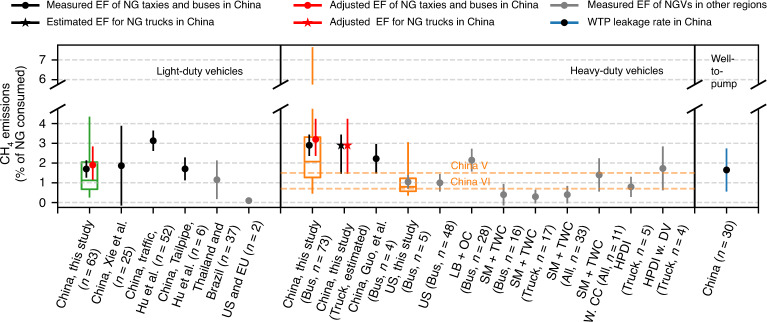
Fuel-specific emission factors as % of NG consumed for light-duty natural gas vehicles, heavy-duty natural gas vehicles, and fuel-specific well-to-pump (WTP) NG leakage rate. The boxes and whiskers for our observations show 5th, 25th, 50th, 75th, and 95th percentiles of the observed EFs. Black dots and bars show the average values and standard errors of corresponding EFs measured in China. Black dots and bars show the average values and standard deviation (S.D.) of corresponding EFs measured in China. Numbers of independent samples (vehicles) used to derive EFs and the standard errors are listed in the labels. Red dots and bars show the venting-emission and seasonality adjusted values of corresponding EFs for China. Gray dots and bars show the average values and standard errors of corresponding EFs measured in other regions. The star and the associated bar show the estimated EF and its uncertainty for heavy-duty NG trucks equipped with a lean-burn engine and oxidation catalyst (determination of the uncertainty can be found in method section). Xie et al. and Guo et al. measured total hydrocarbon (THC) emissions instead of CH_4_^23,52^. We converted their results to CH_4_ emissions assuming 90% of THC is CH_4_ as suggested by Xie et al. and Hu et al.^23,52^. The observed EF for heavy-duty vehicles is 85% higher than the current standard (China V). “LB + OC”, “SM + TWC”, “SM + TWC w. CC”, “HPDI”, and “HPDI w. DV” stand for a lean-burn engine with oxidation catalyst, stoichiometric engine with three-way catalyst, stoichiometric engine with a three-way catalyst with crankcase emissions, high-pressure direct injection (HPDI), and HPDI with dynamic venting emissions. Source data are provided as a Source Data file.

CH_4_ emissions from exhaust and leakage from NG buses and taxis in Baoding and Shijiazhuang were measured by our mobile laboratory equipped with fast-response sensors. We measured 26 h on-road, covering around 600 km in these two cities in June 2014 (details about instruments and spatial coverage can be found in Supplementary Table 3 and Supplementary Fig. 3). The fast-response sensors (10 Hz) allowed the use of the plume-chasing method to measure on-road emissions from vehicles. Several criteria, including sufficient CO_2_ and CH_4_ enhancements, correlations between CH_4_ and CO_2_ and videos recorded on the road, were developed to identify plumes from NGVs. Supplementary Movie 1 provides an example of the on-road measurements. A Gaussian puff model was used to investigate the effectiveness of our method to minimize the influence of the exhaust of nearby vehicles, and the results show our method can significantly reduce interferences caused by the emissions from other vehicles^28^. Using the plume-chasing method, we were able to capture emissions from 73 NG buses and 63 NG taxis during the field campaign. The observed CH_4_ and CO_2_ mixing ratios were used to derive CH_4_:CO_2_ enhancement and emission ratios. The emission ratios were then converted to fuel-specific CH_4_ emission factors. Similar methods have been used to estimate vehicular NH_3_ emissions^29,30^. More details and discussion about the uncertainty of the method can be found in the “Method” section and Supplementary Discussion. Figure 2 shows the on-road fuel-specific CH_4_ EFs (presented as % of NG consumed) derived from CH_4_:CO_2_ emission ratios measured in China as well as previously reported EFs.

Sixty-three NG taxis with clear NGV labels were sampled to represent light-duty NGVs in China, which had an average EF of 1.7 ± 0.5%. The EF is 16 times higher than the values reported for light-duty NGVs in the US and EU (0.10 ± 0.3%), but the EF agrees with the tailpipe CH_4_ EF measured in the exhaust of NG taxis by Hu et al.^23^ (1.7 ± 0.8%). The CH_4_ EF measured from 73 NG buses in China is 2.9 ± 0.5%, which is 90% higher than the CH_4_ limit of the China V standard for heavy-duty vehicles^31^. We were able to distinguish buses powered by liquified natural gas (LNG) and compressed natural gas (CNG) by checking the label of the buses. No statistically significant difference was found between the EFs of LNG buses (39 buses, 2.8 ± 0.4 %) and CNG buses (34 buses, 3.1 ± 0.5 %). The NG buses in these two cities were equipped with LB engine and OC, and they were certified for the China VI and China V standards, respectively. We also observed low NH_3_ emissions from NG buses (Supplementary Fig. 4), consistent with the reported pattern for NGVs with LB engine with OC^32,33^. The observed EF of NG buses is more consistent with the overall on-road CH_4_ EF measured by Hu et al.^23^ (3.0 ± 0.5%) than the observed EF of light-duty NGVs. To validate our method, we conducted additional measurements by following NG buses in Atlantic City, US, in the spring of 2015. The observed EF agrees with previously reported tailpipe CH_4_ emissions for NG buses in the US as well as the CH_4_ emissions used in the GREET model^18^.

### Estimation of CH_4_ emissions from heavy-duty NG trucks

Identifying NG trucks in China was more difficult than NG buses since they were not labeled as clearly as the NG buses. Therefore, we could not derive CH_4_ EF for heavy-duty NG trucks using our observations. Our survey shows that NG trucks certified for China IV and V from the major manufacturers in China are equipped with similar LB engines and OC but with slightly larger displacements than the engines on NG buses (Supplementary Table 4). This type of engine is rarely used on trucks in other countries, and therefore, no CH_4_ EF have been reported for NG trucks equipped with LB engines. Previous studies suggested driving conditions of the vehicles may have larger impacts on CH_4_ emissions rather than the chassis^2,19^. Comparing CH_4_ EFs reported for NG buses and trucks equipped with similar SM engines and TWC, we did not find a significant difference for both the tailpipe and the crankcase CH_4_ emissions (Fig. 2 and Supplementary Table 2)^33–38^. Therefore, the measured CH_4_ EF of NG buses is used to estimate CH_4_ emissions from heavy-duty NG trucks. Since NG trucks may operate on the highway more frequently than NG buses, we assigned a larger error to the lower-bound uncertainty of EFs of NG trucks, which equals to the lower-bound uncertainty of the previously reported CH_4_ EF of LB engines with OC (Fig. 2 and Supplementary Table 2).

### Venting-emission and seasonality adjustment

Because low CO_2_ enhancements and correlations between CH_4_ and CO_2_ mixing ratio enhancements are used to remove impacts from other CH_4_ sources, our method can capture operations related CH_4_ emissions from tailpipes and crankcases but may miss sporadic venting events directly from the on-board fuel tanks that are not fed to the engine. Clark et al.^19^ found these emissions are difficult to be characterized by in-field observations because of the large volume of methane vented in single events and their intermittent nature. Using tank pressure and liquid fuel level (%) differences before and after venting, they estimated the fuel-specific emission rate of these venting events is 0.1% of NG consumed in the US (about 8.4% of total pump-to-wheels CH_4_ emissions for NGVs in the US)^19^. The same emission rate is adopted in our study to account for the venting emissions. Our observations were made in June with an average ambient temperature of 30 °C, which may underestimate CH_4_ emissions during cold seasons, especially for the cold-start emissions. Among the studies reviewed, only two studies reported the cold-start CH_4_ emissions for heavy-duty NGVs at low temperatures. The ratio of cold- and hot-start for CH_4_ EFs at around 0 °C ranges from 1.08 for vehicles with a fuel-specific EF of 11.2% to 2.69 for vehicles with a fuel-specific EF of 0.2% (Supplementary Table 5)^37,39^. To account for the potential impact of cold-start emissions at low temperature, we adjusted the observed EFs using a cold-start/hot-start emission ratio of 1.5 and a weighting factor of 14% for cold-start emissions as listed in the testing procedure for the China VI standard (see “Method” section for details). The adjusted EFs are 1.9 [−0.7, +0.9] %, 3.2 [−0.8, +1.0] %, and 3.2 [−1.7, +1.0] % for NG taxies, heavy-duty NG buses, and heavy-duty NG trucks as shown by the red dots and bars in Fig. 2.

### Technological pathways for the China VI standard

Figure 2 also shows the EFs for SM engines equipped with TWC and the high-pressure direct injection (HPDI) engines. Both have the potential to meet the CH_4_ limit of the China VI standard. However, high CH_4_ emissions from the crankcases of SM engines have been observed as NG could pass through the gaps between the piston rings and the cylinders^19^. When crankcase CH_4_ emissions are considered, it will be difficult for SM engines to meet the China VI standard unless a complicated, closed crankcase ventilation system (CCV) is installed^2^. No crankcase CH_4_ emission has been reported for the HPDI engines, but HPDI engines require venting of the high-pressure fuel to balance NG and diesel fueling pressures, leading to dynamic venting CH_4_ emissions^19^. The dynamic venting CH_4_ emissions could far outweigh the tailpipe CH_4_ emissions during urban operation and could be equivalent to tailpipe emissions during highway operation^19^.

### Well-to-wheels GHG emissions of NGVs in China

Previous studies have estimated the WTW GHG emissions for NGVs in China with limited consideration of CH_4_ emissions from NGVs (see Supplementary Table 6 for studies reviewed)^14,16,22^. Ou et al.^22^ investigated multiple pathways of CNG and LNG in China and reported a WTP leakage rate about 0.6% of NG consumed in the Tsinghua Life Cycle Analysis Model. Huo et al. assumed the technologies in China for production and distribution of CNG and LNG are similar to the ones used in other regions and adopted the rates of 1.93% of NG consumed for extraction and production and 0.007% of NG transported per km via pipeline from the GREET model^16,18^. The difference of WTP GHG emissions between CNG and LNG (1%) is lower than the variation caused by the CH_4_ leakage from pipeline distribution (standard deviation of 7%) since the transport distance ranges from 200 to 4400 km for different provinces. Therefore, the same WTP GHG emission factor (28 ± 6 CO_2eq_ MJ^−1^) and the same WTP CH_4_ leakage rate (1.65 ± 1.05% of NG consumed) are used for both LNG and CNG. The overall WTP leakage rate is about the same as the CH_4_ EF of light-duty NGVs and is 40% lower than the CH_4_ EF of heavy-duty NGVs (Fig. 2).

**Fig. 3:**
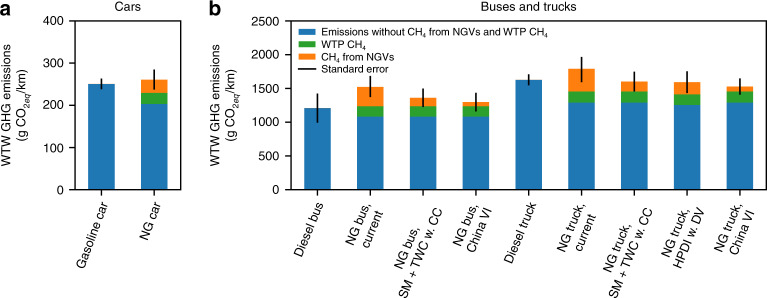
Well-to-wheels GHG emissions of vehicles powered by gasoline, diesel, and NG in China. Panels **a** and **b** show the well-to-wheels GHG emissions for light- and heavy-duty vehicles, respectively, in China. Blue bars show the WTW GHG emissions without CH_4_ contribution. Green and orange bars are the CO_2_ equivalents of WTP CH_4_ emissions and CH_4_ emissions from NGVs (a GWP of 30 over a time-scale of 100 years is used for CH_4_ from fossil fuel consumption according to IPCC AR5)^12^. For cars and buses, NGVs may not bring GHG emission mitigation. NG trucks that meet China VI standard have lower GHG emissions compared to diesel trucks. The black error bars indicate high and low estimates derived using error propagation of uncertainties of multiple input parameters (e.g., life-cycle GHG emissions, CH_4_ emission factors, and fuel consumptions). Uncertainty estimates (standard deviation, S.D.) of individual parameters are listed in Supplementary Tables 1, 6, 7, and 11. Source data are provided as a Source Data file.

The distance-specific WTW GHG EFs for NGVs are derived in this study by combining previously reported upstream GHG EFs, distance-specific fuel consumption, and adjusted CH_4_ EFs of NGVs (shown in Fig. 3). The uncertainty of the national level WTW GHG EF for NGVs in China is large because of the variation in NG transport distance via pipeline (from 200 km to 4400 km). For provincial analysis, as demonstrated by Huo et al.^16^, the uncertainty could be reduced. With the observed CH_4_ emissions, both light-duty NGVs and NG buses are unlikely to reduce GHG emissions compared to their counterparts. For NG buses, the WTW GHG emissions are likely to be higher than diesel buses even if they satisfy the China VI standard CH_4_ limit because of their increased fuel consumption (Supplementary Table 7). Switching from diesel trucks to current generation NG trucks equipped with LB engines and OC as the measured NG buses is likely to increase GHG emissions by 160 [−200, +180] g CO_2eq_ km^−1^. Only the ones operating mostly on the highways in the near-source regions may have lower WTW GHG EF compared to diesel trucks.

For trucks equipped with SM engines and TWC or HPDI engines, the WTW GHG emissions are similar to diesel trucks. It should be noted that the fuel consumption of trucks equipped with SM engines and TWC is assumed to be the same as trucks with LB engines. Operating at lean conditions is an effective way to improve fuel efficiency compared to a pure stoichiometric operation^40^. However, the fuel economy of SM engines can be significantly improved by operating the engine with diluted mixtures through exhaust gas recirculation (EGR) systems, which also can significantly reduce NO_*x*_ emissions^35,40^. Hajbabaei et al.^35^ compared the fuel consumption of a SM engine with an EGR system and two LB engines. They found the SM engine with EGR had very similar fuel consumption compared to the LB engines. For the NG trucks to be certified for the China VI standard, SM engines are likely to be used with an EGR system to be competitive in the market in terms of fuel economy and to be in compliance with the China VI NO_*x*_ emission limit and the China Stage 3 fuel consumption limits^41^. The same fuel consumption was scaled by 0.95 to approximate the fuel consumption of HPDI engines because Thiruvengadam et al.^32^ reported the fuel consumption of HPDI engines was 4% lower than that of SM engines with EGR systems.

If the China VI standard is stringently the enforced with the real-world emissions being the same as the CH_4_ emission limit, switching from diesel trucks to NG trucks will lead to a GHG reduction of 100 ± 150 g CO_2eq_ km^−1^, and upstream CH_4_ leakages will become the limiting factor for lowering the WTW GHG emissions from NGVs in China. Although having real-world emissions in line with certified emission limits is challenging, it has been shown to be technically achievable at least for NO_*x*_ emissions from Euro VI trucks, to which the China VI standard is equivalent^26^.

### CH_4_ emissions from NGVs in China

**Fig. 4:**
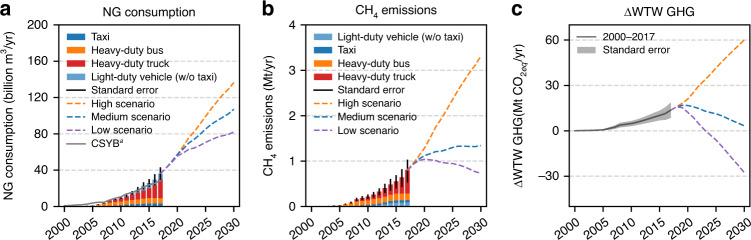
NG consumption, total CH4 emissions from NGVs, and changes of WTW GHG emissions of switching to NGVs in China from 2000 to 2030. Estimated (bars or solid lines) and projected (dashed lines) NG consumption (**a**), total CH_4_ emissions from NGVs (**b**), and changes of WTW GHG emissions of switching to NGVs (**c**) in China from 2000 to 2030. Gray line in **a** shows the reported NG consumption for the Transport, Storage, and Post sector reported in the China Statistical Yearbook (CSYB). When comparing the estimated NG consumption and NG consumption from CSYB, light-duty vehicles (without taxis) should be excluded (light blue bar in **a**). The error bars in **a** and **b** and the gray area in **c** indicate high and low estimates derived using error propagation of uncertainties of multiple input parameters. Uncertainty estimates (standard deviation, S.D.) of individual parameters are listed in Supplementary Table 1, 6, 7, 8, and 11. Source data are provided as a Source Data file.

NG consumption of the Transport, Storage, and Post sector reported in the China Statistical Yearbook (CSYB) does not have the detailed categorical information for estimating CH_4_ emissions from NGVs in China^42^. Therefore, we estimated NG consumption of NG taxis, light-duty NGVs (non-taxi), NG buses, and NG trucks in China as the product of vehicle population (Supplementary Table 1), distance-specific fuel consumption (Supplementary Table 7), and annual mileage traveled (Supplementary Table 8). The four categories are determined based on fuel consumption and emission characteristics and availability of the population data. Figure 4a shows the estimated NG consumption and reported NG consumption in the CSYB^42^. Personal light-duty NGVs (light-duty NGVs except for NG taxis) should be excluded when comparing the estimated NG consumption and the CSYB reported values since fuel consumed by personal vehicles are not included in the Transport, Storage, and Post sector in the CSYB^43^. The sum of NG consumption of NG taxis, buses, and trucks is slightly lower than the CSYB reported consumption because NG consumption of cargo ships is included in the CSYB but not included in our estimates. For 2017, our estimate is closer to the reported consumption of CSYB likely due to the NG shortage in China in the winter of 2017. In 2017, NG buses and trucks consumed about 70% of the total NG consumption of NGVs.

Total CH_4_ emissions and changes in WTW GHG emissions are calculated by multiplying the corresponding emission factors (venting-emission and seasonality adjusted) to the NG consumption (see “Method” section for more details). Figure 4b, c shows the estimated and the projected total CH_4_ emissions from NGVs in China and the changes in WTW GHG emissions of switching to NGVs from gasoline and diesel counterparts for 2000–2030. The annual CH_4_ emissions from NGVs in China increased from 0.0014 [−0.0004, +0.0004] Mt in 2000 to 0.77 [−0.28, +0.22] Mt in 2017. Switching to NGVs has increased the GHG emissions by 83 Mt CO_2eq_ for 2000–2017. More than 80% of CH_4_ emissions from NGVs are emitted by NG buses and trucks in 2017 because of their high fuel consumption and high EFs. Therefore, the implementation of the CH_4_ limit of the China VI standard for heavy-duty vehicles is critical for mitigating future CH_4_ emissions from NGVs.

### Future scenarios

Three scenarios were designed to assess different pathways regarding the implementation of the China VI standard. Table 1 lists the major features of these scenarios. The population estimates are adapted from the projection by Wu et al.^6^, where aggressive electrification for applicable fleets was considered (see Supplementary Table 9 for projected vehicle population for the three scenarios). The fuel consumption of heavy-duty vehicles (both NGVs and conventional gasoline or diesel vehicles) purchased after 2021 is lowered by 15% assuming that the Stage 3 China Fuel Consumption Standard will be implemented successfully^41^.

**Table 1:** Scenarios for projections of future CH_4_ emission and changes in GHG emissions of switching to NGVs.

Scenario	NGV stock	Share of NGV technology	Distance of NG transported
High-emission scenario	Retrofitting is allowed, number of LDNGVs^a^ reaches 8.0 million and remain constant after 2020	Dual-fuel system for LDNGVs	1100 km
	HDNGBs^b^ increase to 0.6 million in 2030	LB^e^ engines with OC^f^ and SCR^g^	
	(20% of city buses, 20% of inter-city buses)		
	HDNGTs^c^ increase to 2.2 million in 2030		
	(25% of heavy-duty trucks)		
Medium-emission scenario	Retrofitting is banned and no new LDNGVs except for taxis after 2020	Dual-fuel system for LDNGVs	1100 km
	HDNGBs increase to 0.48 million in 2030	50% of LB with OC in 2019	
	(20% of city buses, 15% of inter-city buses)	40% of SM^h^ with TWC^i^ in 2019, 80% after 2019	
	HDNGTs increase to 1.76 million in 2030	10% of HPDI^j^ in 2019, 20% after 2019	
	(20% of heavy-duty trucks)^d^		
Low-emission scenario	Retrofitting is banned and no new LDNGVs	Dual-fuel system for LDNGVs	500 km
	HDNGBs increase to 0.36 million in 2030	50% of LB with OC in 2019	
	(20% of city buses, 10% of inter-city buses)	50% of HDNGVs lower than China VI in 2019	
	HDNGTs increase to 1.32 million in 2030	All HDNGVGs lower than China VI after 2019	
	(15% of heavy-duty trucks)		

^a^Light-duty natural gas vehicles.

^b^Heavy-duty natural gas buses.

^c^Heavy-duty natural gas trucks.

^d^The penetration rates for this scenario HDNGBs and HDNGTs are the adapted from PC[2] scenario from Wu et al. ^6^.

^e^Lean burn.

^f^Oxidation catalyst.

^g^Selective catalytic reduction.

^i^Three-way catalyst.

^j^High-pressure direct injection.

The high-emission scenario represents the pathway that retrofitting light-duty vehicles is allowed. In addition, this scenario assumes that the CH_4_ limit of China VI standard is loosely enforced, which has been the case for previous standards as demonstrated here. Although LB engines with OC are considered the last generation technology, they could meet the NO_*x*_ limit of China VI standard if SCR is implemented^11^. If the CH_4_ limit of the China VI standard is loosely implemented, LB engines may dominate the heavy-duty vehicle market because of their advantages in terms of upfront cost, since SM engines require precise air–fuel ratio control strategies and an exhaust gas recirculation system^40^. Under this scenario, annual CH_4_ emissions from NGVs in China would increase to 3.3 Mt, equivalent to 8% of the estimated total anthropogenic CH_4_ emissions and 17% of CH_4_ emissions related to fossil fuel production and consumption in China in 2010^13^. Cumulatively, switching to NGVs from counterparts would increase the WTW GHG emissions by 432 Mt CO_2eq_ from 2020 to 2030 under this scenario (the integrated area under the orange curve in Fig. 4b from 2020 to 2030).

The medium-emission scenario represents the pathway that retrofitting is prohibited, and heavy-duty NGVs sold after 2019 are equipped with SM or HPDI engines. Because of the increased cost, the penetration rate of NGVs is lower than the high-emission scenario. Under this scenario, CH_4_ emissions from NGVs in China would increase at a slower rate, reaching 1.3 Mt in 2030 and the cumulative changes in the WTW GHG emissions from 2020 to 2030 would increase by 117 Mt CO_2eq_.

The low-emission scenario assumes that the EF of the heavy-duty NGVs purchased after 2019 is the same as the CH_4_ limit of China VI standard. The growth of NGVs is assumed to be localized within source regions where NG price is low, and the leakage CH_4_ emissions related to NG distribution are lower than the medium- and high-emission scenarios. The annual CH_4_ emissions from NGVs in China would gradually decrease to 0.7 Mt in 2030 and reduce the WTW GHG emissions by 77 Mt CO_2eq_ cumulatively from 2020 to 2030 under this scenario. Comparing the cumulative WTW GHG changes between the high- and the low-emission scenarios, we find that stringently enforcing the China VI standard for heavy-duty vehicles could generate a GHG reduction of 509 Mt CO_2eq_ for 2020– 2030, equivalent to eliminating GHG emissions of 12 million passenger cars with the current GHG emission level.

## Discussion

NGVs have been promoted in China as a cost-effective alternative to mitigate air pollution and to reduce GHG emissions from vehicles, especially for heavy-duty applications where vehicle electrification remains difficult. Previous studies suggested that the WTW GHG emissions of NGVs were 6–25% lower than gasoline and diesel counterparts, but tailpipe and crankcase CH_4_ emissions from NGVs were mostly ignored^14,16–18^. Hu et al.^23^ sampled on-road CH_4_ emissions from NGVs in China and reported an overall fuel-specific EF of 3.0% and attributed the high on-road CH_4_ emissions to leakage from converted light-duty NGVs. However, our sampling results of 63 NG taxis show similar tailpipe CH_4_ emissions and no significant leakage from light-duty NGVs. Our observations of CH_4_ emissions from 73 NG buses indicate that heavy-duty NGVs contributed more to the high overall on-road CH_4_ emissions in China compared to light-duty NGVs. With the current level of CH_4_ emissions from NGVs, switching to NGVs in China has not brought a reduction of GHG emissions.

With the China VI standard, the heavy-duty transportation sector in China will be “gasified” rapidly because it is less costly for NGVs than diesel and heavy-duty electric vehicles to meet the stringent limits for air pollutant emissions^4^. The rapid growth of heavy-duty NGVs without stringent enforcement of the China VI standard CH_4_ limit, however, would increase CH_4_ emissions as demonstrated by the high-emission scenario. Strictly implementing the China VI standard as in the low-emission scenario will require a closed crankcase ventilation system for SM engines with TWC for heavy-duty NGVs. This pathway would reduce CH_4_ emissions by around 70% in 2030 compared to the high-emission scenario and generate a significant GHG emission reduction, making it a “win-win” option for air quality and climate. Although this measure could lead to an increased price barrier for purchasing NGVs, innovations in the design of engines and after-treatment devices could lower the barrier. For example, catalysts that can remove CH_4_ more effectively from LB engines are being pursued^44–47^. China’s successful implementation of the China VI standard on such a large scale could inspire climatically beneficial NGV development in other regions facing similar challenges, especially in developing countries where natural gas is already considered as an economical alternative for transportation^48,49^.

Substantial uncertainties still exist in the estimates and the projections for CH_4_ and WTW GHG emissions from NGVs in China due to lacking detailed vehicle population and NG consumption data as well as the uncertainties related to CH_4_ EFs of NGVs. With categorized and regional NGV data, the uncertainties for both the CH_4_ emissions and the WTW GHG emissions could be significantly reduced. Despite the large sample size from our campaign, the observed EFs for light-duty NGVs and NG buses may be underestimated because of missing cold-start CH_4_ emissions at low temperature and sporadic venting emissions. In addition, no CH_4_ EF has been reported for current generation NG trucks that are equipped with LB engine and OC and the CH_4_ EF could be different under various driving conditions for the NG trucks. Finally, CH_4_ emissions from NGVs in China in the next decade could vary substantially, depending on the implementation of the China VI standard, as demonstrated in our scenario analyses. Therefore, more observations are needed not only to constrain CH_4_ emissions from current generation NGVs but also to enforce the CH_4_ limit of the China VI standard.

As our measurements demonstrate, there is a discrepancy between real-world CH_4_ emissions and the associated emission limits. For China III, IV, and V, the compliance testing for CH_4_ emissions from NGVs is conducted with the European Transient Cycle (ETC)^31^. The ETC has been criticized for not representing real-world driving conditions since it has relatively high average engine loads over the entire test; consequently, exhaust temperatures during the ETC test are relatively high^50^. Under real-world driving conditions, especially in urban areas, the exhaust temperature from NGVs equipped with LB and OC may be lower than the ideal temperature for CH_4_ removal leading to elevated CH_4_ emissions. This discrepancy highlights the challenges in ensuring that the desired CH_4_ emission levels for NGVs are met in reality and the need for more real-world measurements. For the China VI standard, the compliance testing will be conducted using the World Harmonized Steady-State Cycle (WHSC), which should be more representative of the full range of real-world driving conditions^27^. In addition, starting in 2021, model compliance testing will include a real-world emission test conducted with a portable emissions measurement system (PEMS) attached to the vehicles^27^. However, crankcase and venting emissions may not be captured by the PEMS method. Besides, the results of PEMS testing often show considerable variation because of different vehicle and traffic conditions, as can be seen from EFs measured using PEMS from 25 light-duty NGVs by Xie et al. (Fig. 2), limiting its use to estimate overall emissions^51,52^. Increasing the sample size can reduce the variability but will lead higher costs and difficulties in getting access to the vehicles. Remote sensing or plume-chasing methods, similar to the method used in this study or the method demonstrated by Hu et al.^23^, can provide critical information regarding the fleet-level CH_4_ emissions from NGVs in China to researchers in other fields and the stakeholders. Finally, our method could be applied to other regions as well. For example, a majority of taxis, buses, and autorickshaws in 11 (out of 29) Indian states are powered with CNG due to Indian Supreme Court decisions^53,54^. However, similar to the case in China, CH_4_ emissions from these vehicles have rarely been quantified despite the large fleet size, and EFs developed for other regions were used to investigate climatic impacts of NGVs in India^55^. Our method could be implemented at relatively low cost and, combined with fuel consumption estimates, used to quantify CH_4_ emissions from NGVs in India and total impacts on GHG emissions.

## Method

### Derivation of CH_4_:CO_2_ emission ratio

We used a method similar to Sun et al.^30^ to calculate the CH_4_:CO_2_ emission ratio (in the unit of (ppmv CH_4_) (ppmv CO_2_)^−1^). Observed CH_4_ and CO_2_ mixing ratios were first separated into localized vehicle emission signals (local enhancements) from the urban backgrounds by finding their 2nd percentiles within a 3-min window. Since CH_4_ and CO_2_ are relatively stable (lifetimes about are 12 and 30–95 years for CH_4_ and CO_2_), and we were close to the emission sources, the CH_4_:CO_2_ emission ratio at a given time can be approximated by the slope of orthogonal regression of CH_4_ and CO_2_ enhancements measured within a time window of ±1 s. The short time window was chosen to capture instantaneous changes of CH_4_ emissions. Increasing the time window to ±2.5 s only changed the results by less than 2%.

The plume-chasing method assumes that CH_4_ and CO_2_ are co-emitted from the same sources of interest and are transported and dispersed in the same way. To make sure this assumption is valid, we only used observations when the mobile laboratory was directly following NGVs. With the videos, we were able to distinguish buses powered by liquified natural gas (LNG) and compressed natural gas (CNG) by checking the label of the buses and our on-road videos. No significant difference was observed between the LNG and CNG buses (see Supplementary Table 2 for values), and, therefore, we did not distinguish LNG and CNG buses in the life-cycle analysis and emission scenario analyses.

An example is provided in Supplementary Movie 1, and the associated observations are also shown in Supplementary Fig. 5. We were typically less than 50 m away from the NGVs. Although we did not measure the distance between the NGVs and our mobile laboratory, we used the ΔCO_2_ and ΔCH_4_ thresholds (10 ppmv for ΔCO_2_ and 0.2 ppmv for ΔCH_4_) to determine if the mobile laboratory was within the plume of NGVs. To prevent the influence of the emissions from our mobile platform, we excluded observations with a driving speed smaller than 5 km h^−1^ based on our previous studies^29,56^. As shown in Supplementary Movie 1, there were still vehicles in other lanes even when our mobile laboratory was directly behind the NGVs. Since most of the vehicles were not powered by NG (only 2.6% of the vehicles were powered by NG on a national scale), emissions from other vehicles would contribute to the observed ΔCO_2_, which could potentially lower the estimated emission ratios. To reduce such interference, the correlation between ΔCO_2_ and ΔCH_4_ was used as a criterion to remove observations potentially influenced by other vehicles. Data with *R*^2^ < 0.5 within the ±1 s time window were excluded. The rationale behind this criterion is that high-frequency fluctuations (10 Hz) of CO_2_ and CH_4_ concentrations were caused by turbulent movements of the plumes, and these changes should be correlated if CO_2_ and CH_4_ plumes are emitted from the same source and go thru the same atmospheric transport and dispersion. In addition, using the slope of orthogonal regression between ΔCH_4_ and ΔCO_2_ (instead of the quotient) to estimate enhancement ratio helps to remove the ΔCO_2_ offsets caused by emissions from other vehicles. To investigate the effectiveness of correlation criterion and orthogonal regression, we used a Gaussian puff model (PUFFER) to simulate on-road CO_2_ and CH_4_ concentrations measured by our mobile laboratory (see Supplementary Discussion for more details)^28^. Our results highlight the importance of capturing high-frequency variations. When high-frequency fluctuation is present, which was usually the case for busy roads when our measurements were influenced by other vehicles, no statistically significant difference between the true and the estimated ΔCH_4_:ΔCO_2_ ratios is found, indicating that the combination of R^2^ filtering and orthogonal regression effective in minimizing the interference.

As shown in Supplementary Movie 1, the emission ratio could vary by a factor of three following single bus. This variability is related to driving mode changes and leads to the skewness of observed EFs shown in Fig. 2. Similar variability has been reported by previous studies using the chassis dynamometer method^33,57^. This variability was largely reduced by using fleet-wise mean emission ratios. During our field campaign in China, we had three days of observations and we treated observations from each day as an individual fleet sample. The relative standard deviations of the daily averaged EFs for NG buses and taxis are 3 and 12%. The major source of uncertainty in our method is the plume-identification process. Our results are insensitive to the choice of temporal window for background removal and the thresholds for ΔCO_2_ and R^2^. However, our results are sensitive to the threshold for ΔCH_4_. For example, changing the threshold for *R*^2^ to 0.25 or 0.75 from 0.5 leads to 2–8% changes in the mean ER, much smaller than the impacts of the CH_4_ cutoff (see Supplementary Table 10 for details). In addition, *R*^2^ filtering is less sensitive as the CH_4_ threshold increases. With a CH_4_ threshold of 0.4 ppmv, the daily mean ER changed <2% when increasing *R*^2^ threshold from 0.25 to 0.75. Therefore, we estimated our standard errors of ER related to plume identification as the difference of the results without a cutoff for CH_4_ and the results with a CH_4_ cutoff of 0.4 ppmv. Finally, we estimated the uncertainty of our observation using sample-size weighted uncertainty propagation combining the plume-identification uncertainty and sample uncertainty (see Supplementary Table 10 for details).

### Derivation of the fuel-specific CH_4_ emission factors

The observed fuel-specific CH_4_ emission factor (EF) is defined as the ratio of CH_4_ emission rate and fuel consumption rate. Fuel consumption rate can be estimated by the carbon balance method

1 $${\mathrm{EF}}_{{\mathrm{CH}}_4,{\mathrm{obs}}}^{{\mathrm{fuel}}}(\%\, {\mathrm{of}}\,{\mathrm{NG}}\,{\mathrm{consumed}}) 	=\, \frac{{{\Delta} {\mathrm{CH}}_4}}{{{\Delta} {\mathrm{CO}}_2 + {\Delta} {\mathrm{CH}}_4}} \cdot \frac{{M_{{\mathrm{CH}}_{\mathrm{4}}}}}{{M_{\mathrm{C}}}} \cdot w_{\mathrm{c}} \cdot 100\% \\ 	=\, \frac{{{\mathrm{ER}}}}{{1 + {\mathrm{ER}}}} \cdot \frac{{M_{{\mathrm{CH}}_{\mathrm{4}}}}}{{M_{\mathrm{C}}}} \cdot w_c \cdot 100\%,$$

where ΔCH_4_ and ΔCH_2_ are CH_4_ and CO_2_ enhancements in ppmv, $M_{{\mathrm{CH}}_{\mathrm{4}}}$ and *M*_C_ are the molar weights of CH_4_ and carbon, *w*_c_ = 0.75 is the carbon content of NG, and ER is the emission ratio. Since $w_{\mathrm{c}}\,M_{{\mathrm{CH}}_4}/M_{\mathrm{C}} = 1$ for CH_4_ emissions from NGVs, Eq. (1) can be simplified as:

2 $${\mathrm{EF}}_{{\mathrm{CH}}_4,{\mathrm{obs}}}^{{\mathrm{fuel}}}(\% \,{\mathrm{of}}\,{\mathrm{NG}}\,{\mathrm{consumed}}) = \frac{{{\mathrm{ER}}}}{{1 + {\mathrm{ER}}}} \cdot 100\%.$$

CH_4_ emissions and CO_2_ emissions from other studies are converted to fuel-specific emission factor using the same method when they are not provided (Supplementary Table 2). CO was neglected in this study to be consistent with past literature and because CO emissions are often not available. Adding the observed CO concentrations to Eq. (1) would decrease the fuel-specific CH_4_ emission factors by 2%. The fuel-specific emission factors that include CO emissions can be found in Supplementary Table 2.

### Venting-emission and seasonality adjusted fuel-specific CH_4_ emission factors

The observed EFs are adjusted to account for venting emissions and seasonality as

3 $${\mathrm{EF}}_{{\mathrm{CH}}_{\mathrm{4}}{\mathrm{,adj}}}^{{\mathrm{fuel}}} = {\mathrm{EF}}_{{\mathrm{CH}}_{\mathrm{4}}{\mathrm{,obs}}}^{{\mathrm{fuel}}} \cdot (1.5 \times 0.14 + 1 \times 0.86) + 0.1\%,$$

where cold-start emissions are estimated using a cold-start/hot-start emission ratio of 1.5. The cold-start/hot-start emission ratio is determined as the average cold-start/hot-start emission ratio for an EF near 3% of fuel consumed (Supplementary Table 5). Cold-start and hot-start are averaged with weighting factors of 14% and 86% (adapted from the testing procedure listed in the China VI standard), respectively^27^. Venting CH_4_ emissions from the on-board tank are not related to the combustion process and are not co-emitted with CO_2_ emissions. Therefore, these emissions were not captured by our method and should be compensated. Therefore, 0.1% of NG consumed is added to compensate the venting EF as reported by Clark et al.^19^. The lower-bound uncertainty remains unchanged as the lower-bound uncertainty of the observed ERs to account for the possibility that ambient temperature and venting events have no impact on CH_4_ emissions. The upper-bound uncertainty (${\mathrm{UBEF}}_{{\mathrm{CH}}_{\mathrm{4}}{\mathrm{,adj}}}^{{\mathrm{fuel}}}$) is estimated as

4 $${\mathrm{UBEF}}_{{\mathrm{CH}}_{\mathrm{4}}{\mathrm{,adj}}}^{{\mathrm{fuel}}} = {\mathrm{UBEF}}_{{\mathrm{CH}}_{\mathrm{4}}{\mathrm{,obs}}}^{{\mathrm{fuel}}} \cdot (2.7 \times 0.14 + 1 \times 0.86) + 0.1\%,$$

to account for the possibility of large temperature impact on CH_4_ emissions as reported by Olofsson et al.^37^ for a lower EF.

### Estimation of WTW GHG emissions

The GHG emissions considered in this study include CO_2_, CH_4_, and nitrous oxide (N_2_O). The CO_2_ equivalents for CH_4_ and N_2_O were calculated with 100-year GWPs of 30 and 268 for fossil fuel combustion, respectively^12^. Fuel life-cycle analyses have been conducted for gasoline, diesel and NG in the transportation sector in China, and the reported GHG emissions per fuel (MJ) consumed are listed in Supplementary Table 6. Vehicle CH_4_ emissions from NGVs were largely neglected in previous studies and were included here. Therefore, WTW GHG emissions per km traveled from NGVs can be estimated as

5 $${\mathrm{WTW}}\,{\mathrm{GHG}} = {\mathrm{FC}} \cdot \left( {{\mathrm{GEF}} + {\mathrm{EF}}_{{\mathrm{CH}}_{\mathrm{4}}{\mathrm{,adj}}}^{{\mathrm{fuel}}} \cdot \frac{{M_{{\mathrm{CH}}_{\mathrm{4}}}}}{{M_{{\mathrm{CO}}_{\mathrm{2}}}}}{\mathrm{EF}}_{{\mathrm{CO}}_{\mathrm{2}}}^{{\mathrm{energy}}} \cdot {\mathrm{GWP}}_{{\mathrm{CH}}_{\mathrm{4}}}} \right),$$

where FC is the fuel consumption (MJ km^−1^), *GEF* is the previously reported fuel life-cycle GHG emissions (kg CO_2eq_ MJ^−1^), $M_{{\mathrm{CH}}_{\mathrm{4}}}$ and *M*_C_ are the molar weights of CH_4_ and CO_2_, ${\mathrm{EF}}_{{\mathrm{CO}}_{\mathrm{2}}}^{{\mathrm{energy}}} = 55.72$ (kg CO_2_ MJ^−1^) is the CO_2_ emission factor per MJ natural gas consumed recommended by the IPCC^58^, and ${\mathrm{GWP}}_{{\mathrm{CH}}_{\mathrm{4}}} = 30$ is the 100-year GWP for CH_4_. Fuel consumption can be found in Supplementary Table 7. The ${\mathrm{EF}}_{{\mathrm{CO}}_{\mathrm{2}}}^{{\mathrm{energy}}}$ includes CO_2_ oxidized from the escaped fuel carbons (in the form of CO, CH_4_, and other hydrocarbons)^58^. Carbon emissions in the form soot is ignored since the reported soot emissions are extremely low for NGVs (<1 × 10^−3^%)^59^. In addition, we also assigned 5% uncertainty to ${\mathrm{EF}}_{{\mathrm{CO}}_{\mathrm{2}}}^{{\mathrm{energy}}}$ to account for potential changes in the oxidation rate as well as potential variation in the composition of natural gas (previously reported value ranges from 55.54 to 57.7 kg CO_2_ MJ^−1^ for China)^14,17^.

### Compilation of the bottom-up CH_4_ emission inventory for NGVs in China

The total CH_4_ emissions from NGVs in China are estimated by multiplying the category-specific ERs to the corresponding CO_2_ emissions. The CO_2_ emissions from NGVs are calculated using the estimated NG consumption based on vehicle population and annual mileage traveled in each category. The estimated NG consumption is consistent with the official statistics (Fig. 3a). Four categories are defined: NG taxis, private light-duty NGVs, NG buses, and heavy-duty NG trucks. We estimate CH_4_ emissions from NG taxis and light-duty NGVs separately because of the significant difference between their annual mileage traveled (Supplementary Table 8). Therefore, CH_4_ emissions from NGVs can be described as

6 $${\mathrm{Emis}}_{{\mathrm{CH}}_{\mathrm{4}}} = \mathop {\sum}\limits_{i = 1}^4 {\mathop {\sum}\limits_{y = 1}^{y_{{\mathrm{retire}}}} {{\mathrm{EF}}_{{\mathrm{CH}}_{\mathrm{4}}{\mathrm{,adj,}}i}^{{\mathrm{fuel}}}} } \cdot \frac{{M_{{\mathrm{CH}}_{\mathrm{4}}}}}{{M_{{\mathrm{CO}}_{\mathrm{2}}}}} \cdot {\mathrm{AMT}} \cdot {\mathrm{FC}}_i \cdot {\mathrm{EF}}_{{\mathrm{CO}}_{\mathrm{2}}}^{{\mathrm{energy}}} \cdot {\mathrm{VP}}_{i,y},$$

where *i* is the vehicle category, *y* is the age of the vehicles (compulsory retirement age (*y*_*r*_) ranges from 8 to 15, see Supplementary Table 9 for more details), AMT_*i,y*_ is the annual mileage traveled by vehicles in category *i* with age *y* (km, see Supplementary Table 9 for details), FC_*i*_ is the fuel consumption for category *i* (MJ km^−1^, see Supplementary Table 7), ${\mathrm{EF}}_{{\mathrm{CO}}_{\mathrm{2}}}^{{\mathrm{energy}}}$ is the energy-specific CO_2_ emission factor^58^, and VP_*i,y*_ is the vehicle population for category *i* with age *y*. Uncertainty of annual emissions are calculated using uncertainty propagation, and relative standard errors for these parameters can be found in Supplementary Table 11.

### Calculating GHG emission changes of switching to NGVs

The GHG emission changes of switching to NGVs are calculated similar to total CH_4_ emissions from NGVs

7 $${\Delta} {\mathrm{WTW}}\,{\mathrm{GHG}} = \mathop {\sum}\limits_{i = 1}^4 {\mathop {\sum}\limits_{y = 1}^{y_{retire}} {({\mathrm{WTW GHG}}_{{\mathrm{NG}},i} - {\mathrm{WTW GHG}}_{{\mathrm{CG}}/{\mathrm{CD}},i}) \cdot } } {\mathrm{AMT}}_{i,y} \cdot {\mathrm{VP}}_{i,y},$$

where WTW GHG_NG,*I*_ is the WTW GHG emission of NGVs in category *i*, and WTW GHG_*CG/CD,I*_ is the WTW GHG emission of conventional gasoline or diesel vehicles in category *i*.

### Reporting summary

Further information on research design is available in the Nature Research Reporting Summary linked to this article.

## Supplementary information

Supplementary Information

Reporting Summary

Description of Additional Supplementary Files

Supplementary Data 1

Supplementary Movie 1

## Data Availability

Time series of raw observations used for emission ratio calculations (10 Hz), time series of ΔCH_4_:ΔCO_2_ and ΔNH_3_:ΔCO_2_, and their determination coefficients (*R*^2^) are included in Supplementary Data 1. The data are also available from DataSpace at Princeton University [10.34770/t009-7064]. Other data related to emission calculation are listed in the main text or in Supplementary Information. Source data are provided with this paper.

## Source data

Source Data

## Code availability

The source codes for calculating enhancement ratios and *R*^2^ and for Gaussian puff model are provided within Supplementary Data 1. The codes are also archived in https://github.com/dp7-PU/CH4_from_NGV_in_China.
