# Supplementary material for: Methane emissions from natural gas vehicles in China
Source: Nat Commun. 2020 Sep 11;11:4588. doi: 10.1038/s41467-020-18141-0 (PMC7486943; doi:10.1038/s41467-020-18141-0)
Supplement: Supplementary file 1 — Supplementary Information [file 41467_2020_18141_MOESM1_ESM.pdf]

Supplementary Information for

Methane Emissions from Natural Gas Vehicles in China

Pan et al.

Supplementary Figures

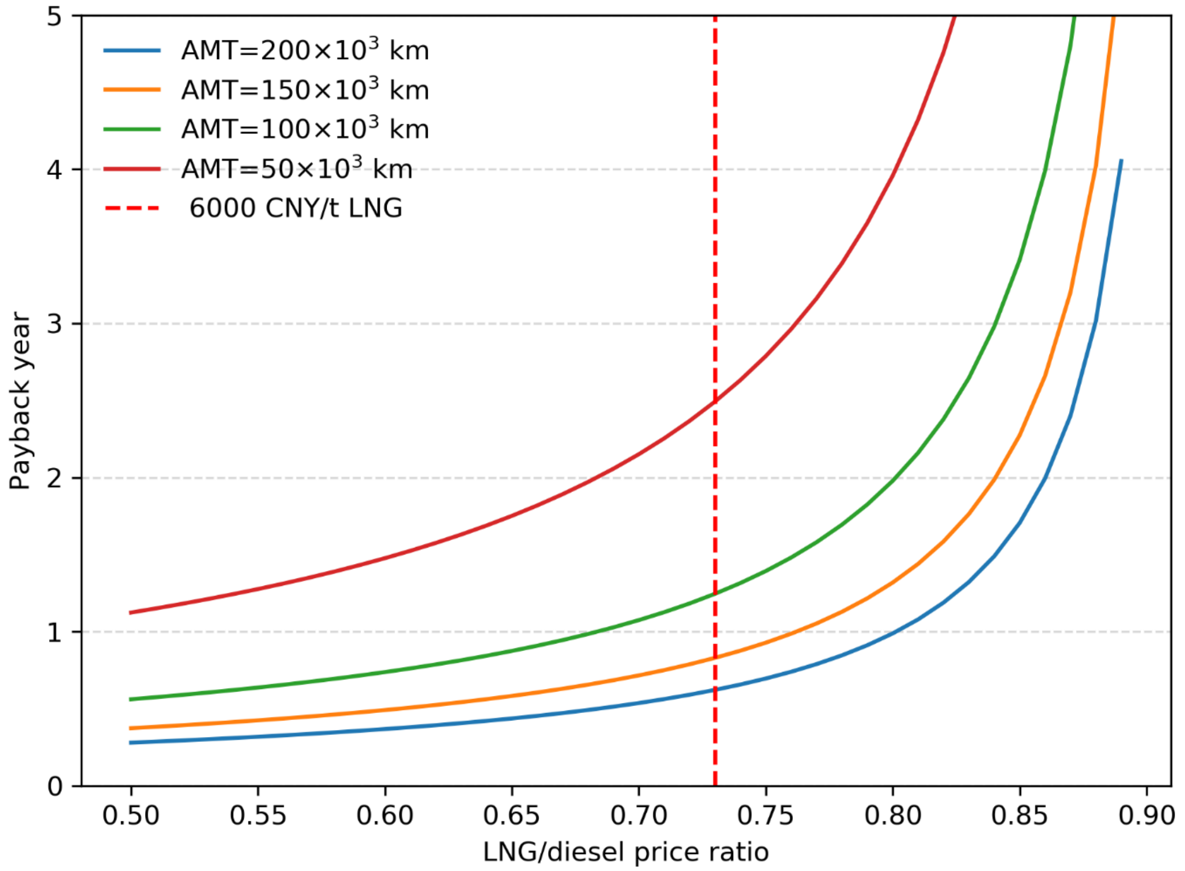

S

Supplementary Figure 1. Payback times of switching from heavy-duty diesel truck to heavy-duty LNG truck, assuming: 1) Vehicle price difference is 50000 CNY; 2) Fuel economies are as described in Table S4; 3) Diesel price at 6.5 CNY/L; 4) Discount rate and interest are not considered. LNG price has been lower than 6000 CNY/ton in China except for the winter of 2017 (Pricing Supervision Center, National Development and Reform Commission of the People's Republic of China, http://jgjc.ndrc.gov.cn/Detail.aspx?newsId=6362&TId=706, accessed in Dec., 2018). Multiple annual mileage traveled (AMT) scenarios are considered. Source data are provided in the Source Data file.

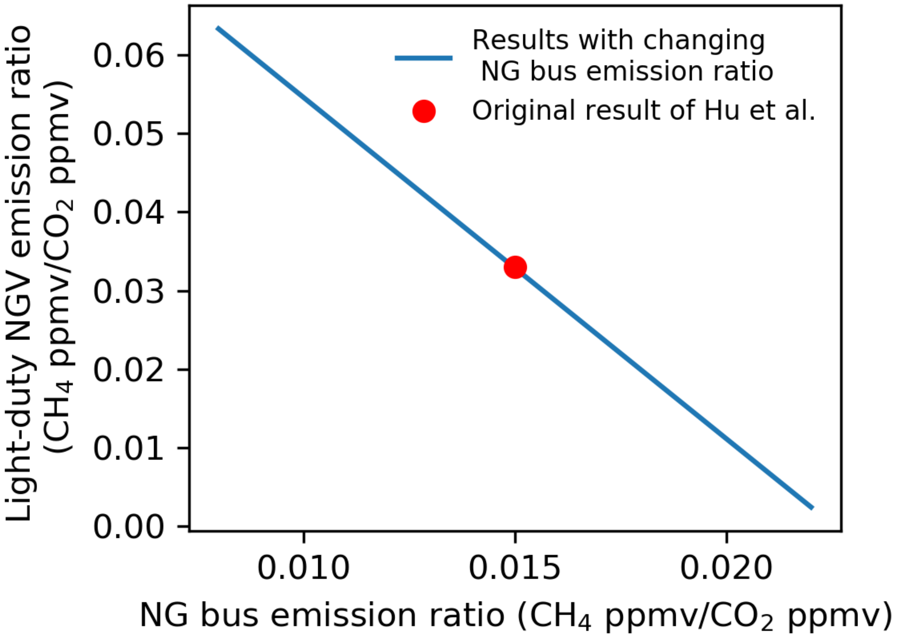

Supplementary Figure 2. Sensitivity of the light-duty NGV emission ratio defined by Hu et al. to the NG bus emission ratio[2]. Hu et al. (3) used the regression between the observed traffic emission ratio ($\boldsymbol{TER}$) and the observed NGV fraction ($\boldsymbol{f}$) to determine light-duty NGV emission ratio ($\boldsymbol{R_2}$):

$$\boldsymbol{TER = \frac{f \cdot R_1 + (1-f) \cdot R_2 \cdot \left(E_{c,1}/E_{c,2}\right)}{f + (1-f) \cdot \left(E_{c,1}/E_{c,2}\right)}}$$

where $\boldsymbol{R_2 = 0.992 \times 10^{-3}}$ ppmv CH_4/ppmv CO_2 is the emission ratio of conventional vehicles, $\boldsymbol{E_{c,1}/E_{c,2} = 1.164}$ is ratio of the CO_2 emission factors of light-duty NGVs and conventional vehicles. This regression does not consider emissions from NG buses. To test the impact of NG bus emissions, Hu et al. modified the regression by dividing NGVs into two groups and assigned an emission ratio of 0.015 to NG buses ($\boldsymbol{R_b}$). The modified equation for the regression can be expressed as

$$TER = \frac{f/2 \cdot R_1' + f/2 \cdot R_b \cdot (E_{c,b}/E_{c,2}) + (1-\mathrm{f}) \cdot \mathrm{R_2} \cdot \left(\mathrm{E_{c,1}/E_{c,2}}\right)}{f/2 + f/2 \cdot E_{c,b} + (1-\mathrm{f}) \cdot \left(\mathrm{E_{c,1}/E_{c,2}}\right)}$$

where $\mathrm{E_{c,1}/E_{c,2}} = 4.35$ is ratio of the CO_2 emission factors of NG buses and conventional vehicles. When R_b is assigned as 0.015, R_1' does not differ significantly compared to $R_1 = 0.031$. However, the uncertainty range (standard deviation of reviewed values by Hu et al. (3)) for R_b is 0.0079 to 0.0221. Within this range, R_1' can change by more 90% from 7% to 192%, which means the light-duty NG emission ratio is very sensitive to the choice of NG bus emission ratio. Source data are provided in the Source Data file.

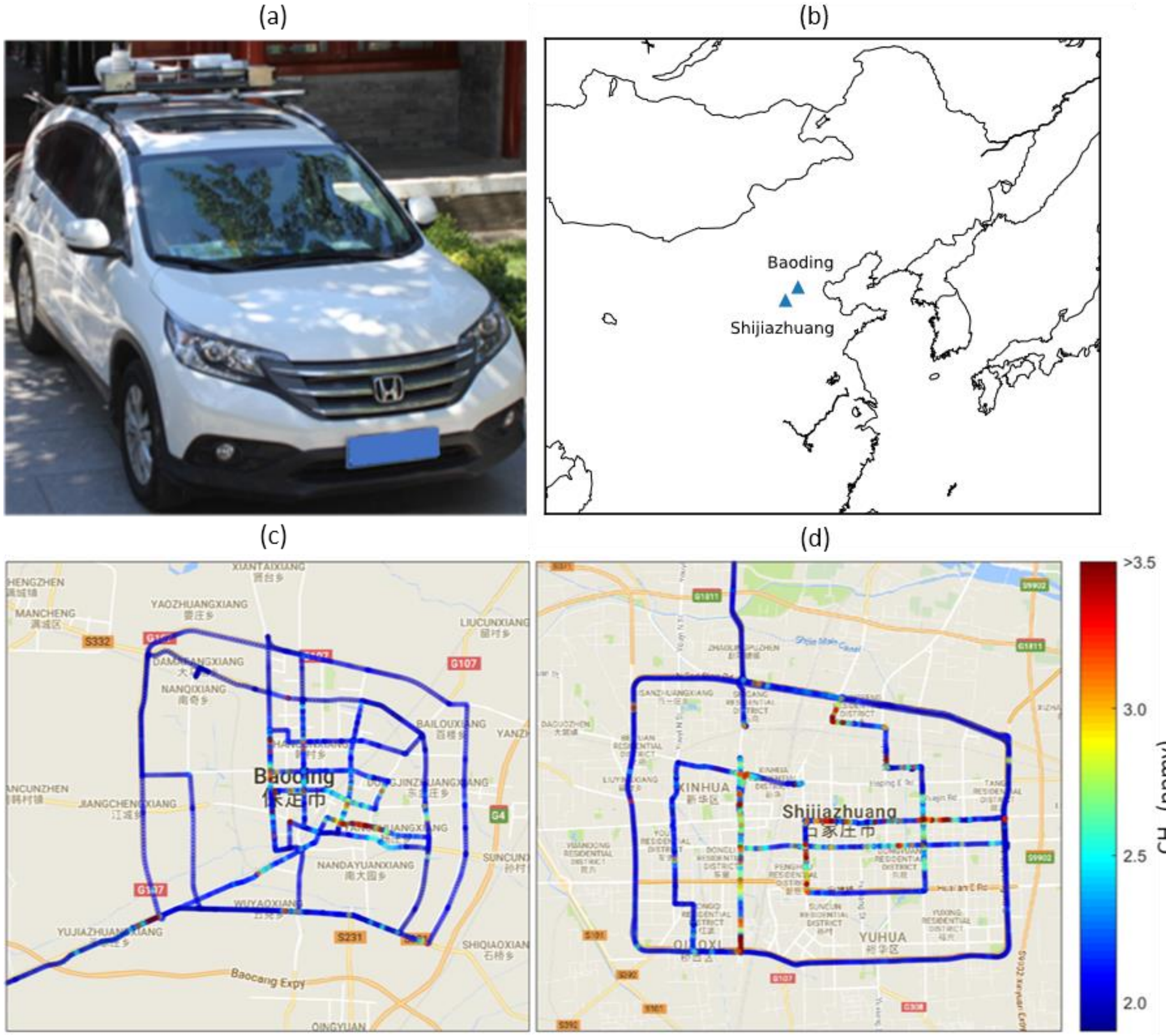

Supplementary Figure 3. (a) Mobile platform; (b) Location of Baoding and Shijiazhuang; (c) CH_4 concentrations observed in Baoding; (d) CH_4 concentration observed in Shijiazhuang. Some streets were sampled multiple times.

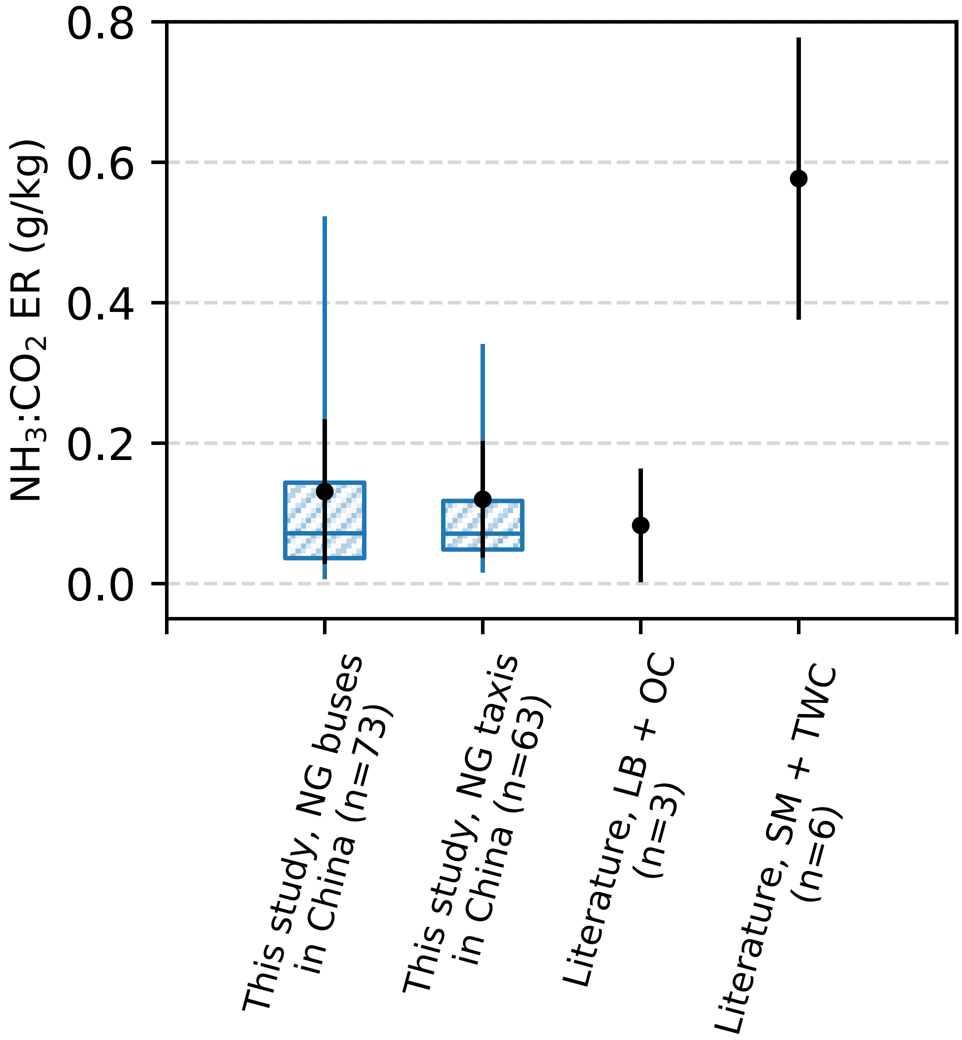

Supplementary Figure 4. NH_3:CO_2 emission ratios. The boxes and whiskers for our observations show 5th, 25th, 50th, 75th and 95th percentiles of the observed EFs. Literature NH_3 emissions for lean-burn engines with oxidation catalyst (LB+OC) and stoichiometric engines with three-way catalyst (SM+TWC) were obtained from Hajbabaei et al., 2013(12), Karavalsis et al., 2016(13), and Thiruvengadam et al., 2016(20). The numbers of independent samples (vehicles) are listed in the labels.

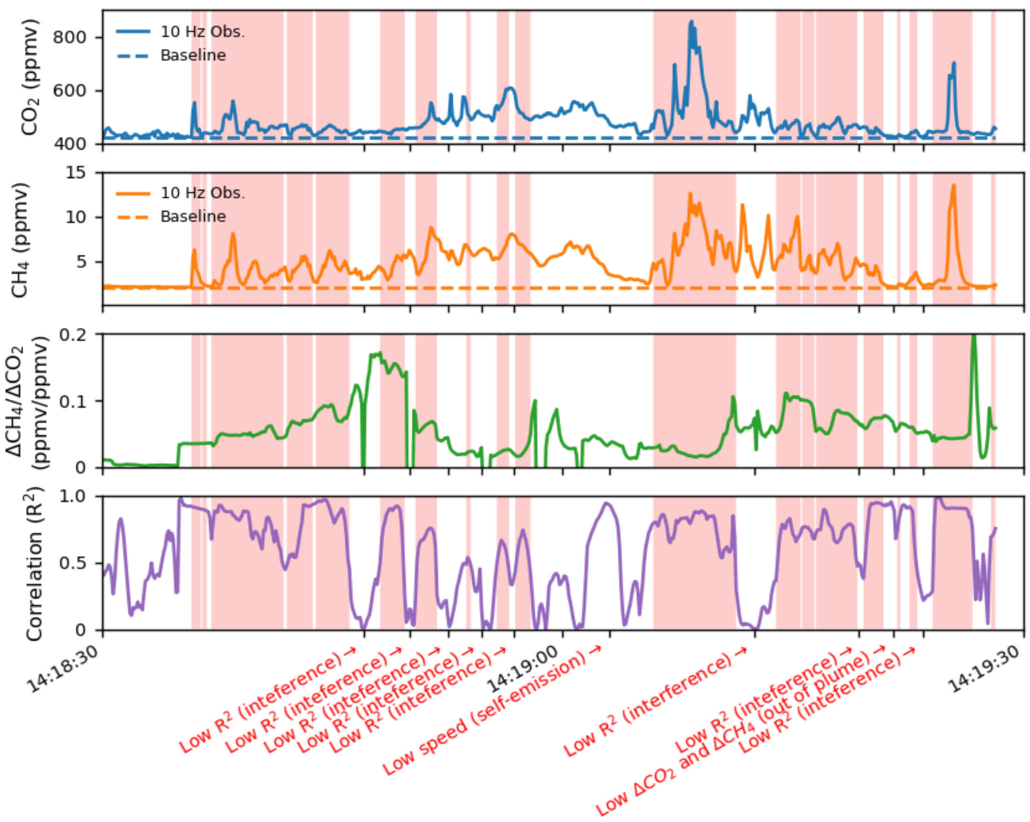

Supplementary Figure 5. An example of observed CO_2 concentrations, CH_4 concentrations, $\Delta CH_4/\Delta CO_2$ ratios, and R^2. Periods met all the criteria are colored red. Reasons of exclusion are labeled on x-axis. Source data are provided in the data archive.

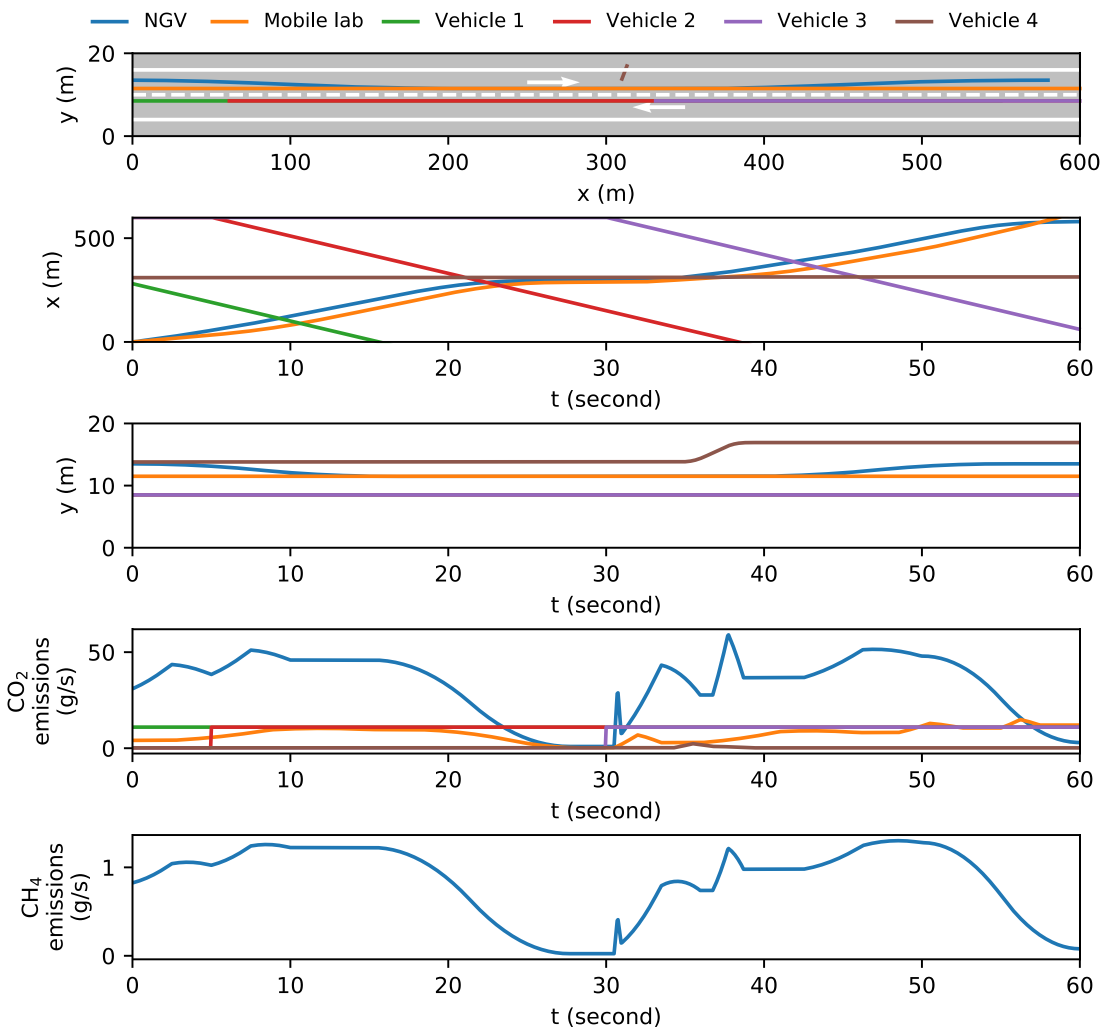

Supplementary Figure 6. Trajectories and emissions of the vehicles. Six vehicles are included in the model. Vehicle 1 – 3 are on the opposite lane of the NGV and the mobile laboratory. Vehicle 4 appears briefly between the NGV and the mobile laboratory. The bottom two panels show CO_2 emissions from NGV, mobile laboratory, and Vehicle 1 – 4 and CH_4 emissions from the NGV.

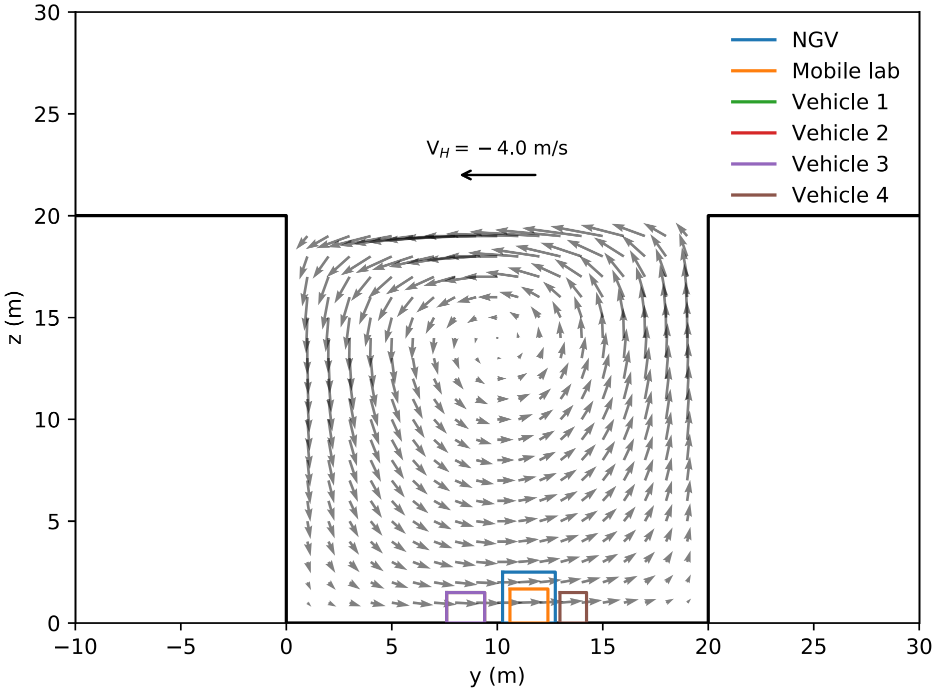

Supplementary Figure 7. Flow pattern along z-y plane caused by wind above the street canyon. Cross sections of the vehicles are also shown in the figure.

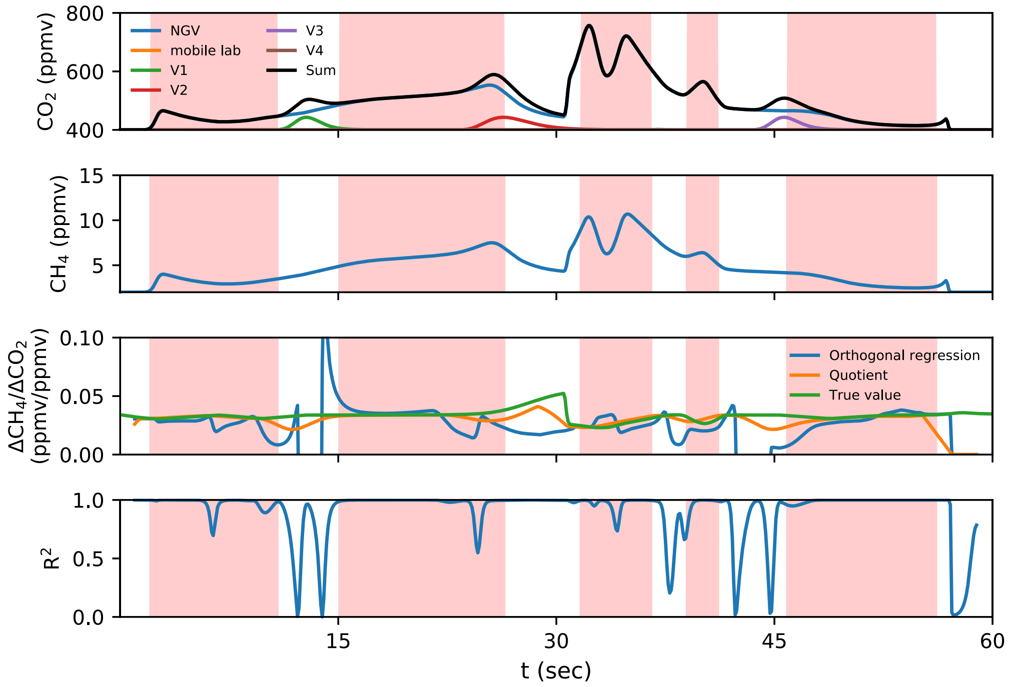

Supplementary Figure 8. Simulated CO_2 and CH_4 concentrations, ΔCH_4: ΔCO_2 ratio, and R^2 using PUFFER. The periods satisfied the plume identification criteria are colored red.

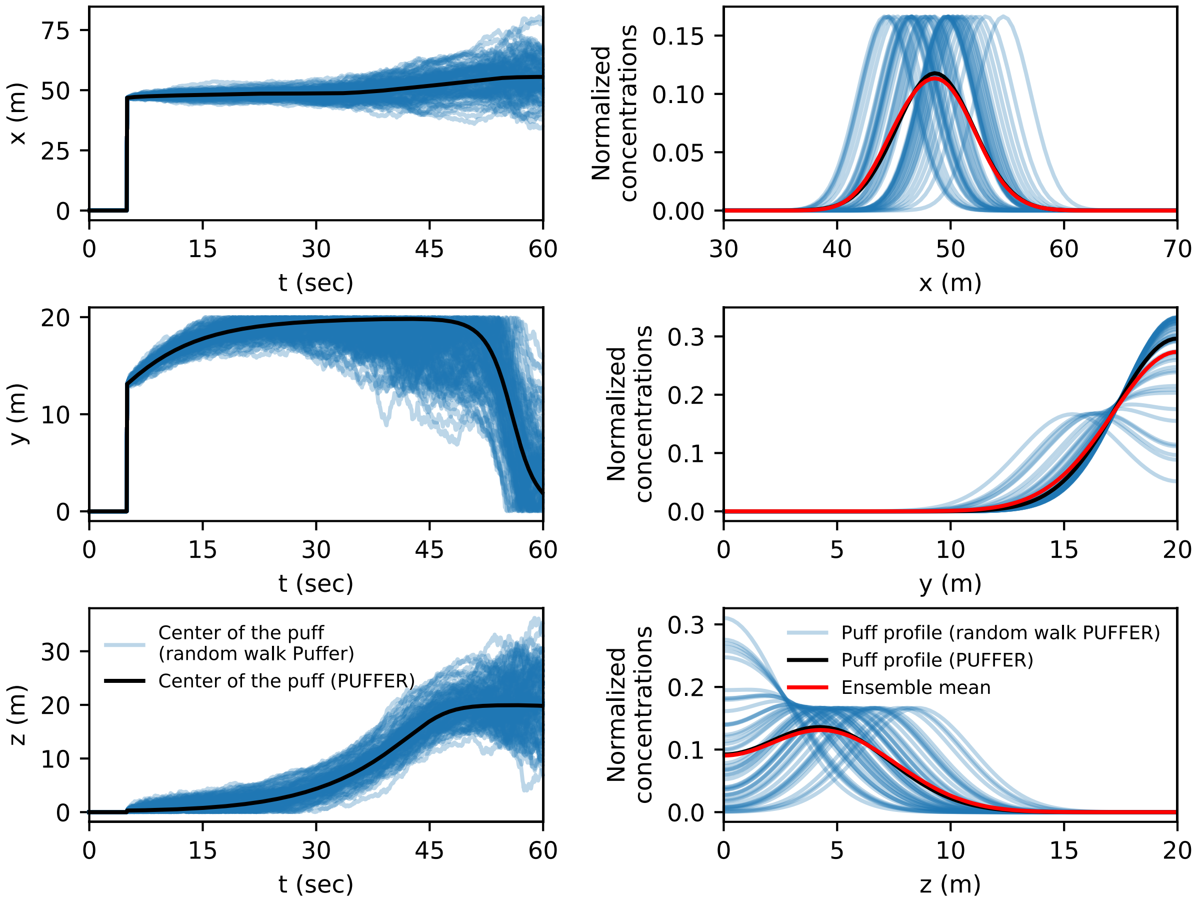

Supplementary Figure 9. Trajectories of a puff emitted by the NGV at t=5 second. The black and blue lines are results from PUFFER and random walk PUFFER, respectively. 100 simulations were conducted to generate the trajectories for random walk PUFFER. The three panels on the right show profiles and ensemble profiles of PUFFER and random walk PUFFER along x-, y-, and z-axes at t=30 second.

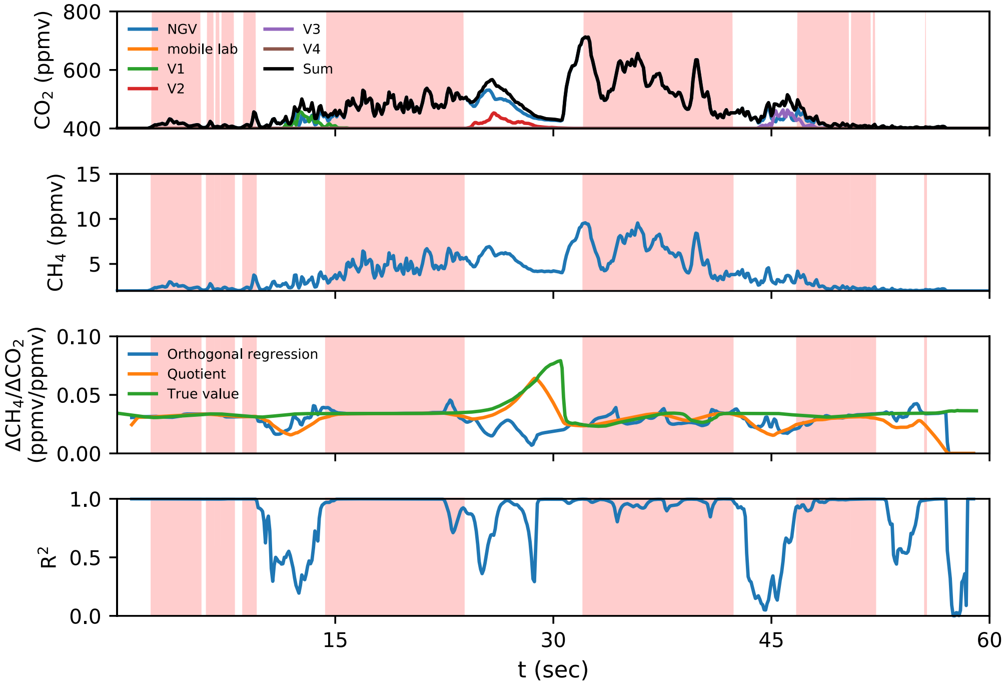

Supplementary Figure 10. Simulated CO_2 and CH_4 concentrations, ΔCH_4: ΔCO_2 ratio, and R^2 using PUFFER. The periods satisfied the plume identification criteria are colored red.

Supplementary Tables

Supplementary Table 1. Population of natural gas vehicle in China from 2000-2017.

Year	Total NGV	Light-duty NGVs without Taxi	Heavy-duty NG bus	Heavy-duty NG truck	NG taxi
2000	6000 [a]	3000 [a]	2000 [a]	0 [a]	1000 [b]
2001	36000 [a]	21000 [a]	8000 [a]	0 [a]	7000 [b]
2002	52600 [a]	30450 [a]	12000 [a]	0 [a]	10150 [b]
2003	69300 [a]	39975 [a]	16000 [a]	0 [a]	13325 [b]
2004	118000 [a]	71175 [a]	23100 [a]	0 [a]	23725 [b]
2005	188000 [a]	116700 [a]	32400 [a]	0 [a]	38900 [b]
2006	307000 [a]	175800 [a]	72600 [a]	0 [a]	58600 [b]
2007	496000 [a]	286609 [a]	112755 [a]	1100 [a]	95536 [b]
2008	700000 [a]	390000 [a]	150000 [a]	30000 [a]	130000 [b]
2009	916000 [a]	534188 [a]	163750 [a]	40000 [a]	178063 [b]
2010	1127000 [a]	648558 [a]	177500 [a]	50000 [a]	250942 [c]
2011	1526000 [a]	929854 [a]	205000 [a]	61905 [a]	329241 [c]
2012	2162000 [a]	1456925 [a]	223570 [a]	88929 [a]	392576 [c]
2013	3327500 [d]	2526433 [e]	242140 [f]	115953 [f]	442975 [c]
2014	4606000 [d]	3661535 [e]	260710 [f]	142976 [f]	540779 [c]
2015	5190000 [g]	4152000 [e]	280000 [g]	170000 [g]	618515 [g]
2016	5576000 [d]	4347250 [e]	285000 [f]	247500 [f]	660258 [f]
2017	6080000 [g]	4763000 [e]	290000 [g]	325000 [g]	702000 [g]

[a] Natural and Bio Gas Vehicle Association, http://www.ngvaeurope.eu/ngv-statistics-june-2013-update, accessed in 2016/07; [b] Estimated as applying the fraction of NG taxis in light-duty NGVs in 2010 when official statistics became available; [c] Ministry of Transport of the People's Republic of China, China Transport Statistical Yearbook 2010 - 2014(1); [d] NGV Global, Current Natural Gas Vehicle Statistics, http://www.iangv.org/current-ngv-stats/, accessed in 2018/03; [e] Estimated by subtracting stocks of vehicle in other categories; [f] Linear interpolated from years with reliable data sources; [g] Sichuan Clean-Energy Information Association, http://scqjqc.cn/lanmu/shuju/ (in Chinese);

Supplementary Table 2. Comparison of emission ratios and fuel-specific emission factors[a].

Source	Number of independent samples	CH_4 emission factor or concentration	CO_2 emission factor or concentration	CH_4:CO_2 emission ratio (ppmv/ppmv)	Fuel specific emission factor (%)
**Light-duty natural gas vehicles in China**					
Xie et al., retrofitted, 2011 (2)[b]	8	1.3 ± 1.4 g/km	164 ± 30 g/km	0.02 ± 0.02	2 ± 2
Xie et al., OEM, 2011 (2)[b]	17	1.2 ± 1.4 g/km	161 ± 26 g/km	0.02 ± 0.02	2 ± 2
Hu et al., traffic, 2018 (3)	52			0.031 ± 0.005	3.0 ± 0.5
Hu et al., tailpipe, 2018 (3)	6			0.017 ± 0.008	1.7 ± 0.8
This study (observed with CO)	63			0.0165 ± 0.0051	1.6 ± 0.5
This study (observed)	63			0.016 ± 0.0052	1.7 ± 0.5
This study (venting-emission and seasonality adjusted)				0.019 [-0.007, 0.009]	1.9 [-0.7, 0.9]
**Light-duty natural gas vehicles in US and EU**					
Karavalakis et al., 2012 (4)	1	0.06 ± 0.01 g/km	192 ± 1 g/km	0.0008 ± 0.00013	0.08 ± 0.01
Bielaczyc et al., 2014 (5)	1	0.067 g/km	133.1 g/km	0.0011	0.11
Average[c]				0.001 ± 0.0003	0.10 ± 0.03
GREET Model (6)					0.09
**Light-duty natural gas vehicles in Thailand and Brazil**					
Lima et al., 2010 (7)	25	178 ± 128 ppmv	1.8 ± 0.5 %	0.01 ± 0.01	1 ± 1
Nilrit et al., 2013 (8)	12	0.8 ± 0.4 g/km	164 ± 5 g/km	0.013 ± 0.07	1.3 ± 0.7
Average[c]				0.012 ± 0.009	1.2 ± 0.9
**Natural gas buses in China**					
Guo et al., 2014 (9)[b]	4	7.1 ± 1.7 g/km	895 ± 71 g/km	0.022 ± 0.007	2.2 ± 0.8
Yue et al., 2016 (10)[b,d]	1	5.0 g/km	500 g/km	0.028	2.8
This study (observed, with CO)	73			0.0294 ± 0.0046	2.9 ± 0.4
This study (observed)	73			0.0301 ± 0.0047	2.9 ± 0.5
This study (venting-emission and seasonality adjusted)				0.033 [-0.008, 0.011]	3.2 [-0.8, 1.0]
This study (observed), LNG	39			0.028 ± 0.004	2.8 ± 0.4
This study (observed), CNG	34			0.032 ± 0.005	3.1 ± 0.5
**Natural gas trucks in China**					
This study (estimated)				0.030 [-0.015,0.005]	2.9 [-1.4,0.5]
This study (venting-emission and seasonality adjusted)				0.033 [-0.018,0.011]	3.2 [-1.7,1.0]
**Natural gas buses in US**					
Yoon et al., 2013 (11)	31	7 ± 3 g/mile	1868 ± 295 g/mile	0.010 ± 0.003	1 ± 0.3
Hajbabaei et al., 2013 (12)	4	10 ± 4 g/mile	1753 ± 117 g/mile	0.016 ± 0.006	1.5 ± 0.6
Karavalakis et al., 2016 (13)	1	11 ± 2 g/mile	1658 ± 35 g/mile	0.018 ± 0.003	1.8 ± 0.3
Clark et al., 2017 (14)	12			0.0095 ± 0.0026	0.95 ± 0.26
Average[c]				0.011 ± 0.004	1.0 ± 0.4

GREET Model (6)				0.013	1.3
This study	5			0.010 ± 0.003	1.0 ± 0.3
**CH_4 limits of China III, VI, V, VI for heavy-duty NG engines**					
China III (15)		1.6 g/kWh	199.8 g/kWh	0.022	2.2
China IV (15)		1.1 g/kWh	199.8 g/kWh	0.015	1.5
China V (15)		1.1 g/kWh	199.8 g/kWh	0.015	1.5
China VI (16)		0.5 g/kWh	199.8 g/kWh	0.007	0.7
**Lean-burn engines with oxidation catalyst**					
Hesterberg et al., 2008 (Bus) (17)	21	20.98 ± 3.90 g/mile	2558 ± 215 g/mile	0.023 ± 0.005	2.2 ± 0.5
Yoon et al., 2013 (Bus) (11)	4	9.80 ± 2.26 g/mile	2074 ± 50 g/mile	0.013 ± 0.003	1.3 ± 0.3
Hajbabaei et al., 2013 (Bus) (12)	2	11.9 ± 3.1 g/mile	1714 ± 63 g/mile	0.019 ± 0.005	1.9 ± 0.5
Karavalakis et al., 2016 (Bus) (13)	1	11 ± 2 g/mile	1658 ± 35 g/mile	0.018 ± 0.003	1.8 ± 0.3
Average				0.022 ± 0.005	2.1 ± 0.5
**Stoichiometric engines with three-way catalyst**					
Hesterberg et al., 2008 (Bus) (17)	8	2.8 ± 8.9 g/mile	2291 ± 372 g/mile	0.003 ± 0.006	0.3 ± 0.6
Nylund et al., 2012 (Bus) (18)	1	0.26 g/km	1183 g/km	0.0006	0.06
Yoon et al., 2013 (Bus) (11)	2	3.5 ± 0.4 g/mile	1709 ± 23 g/mile	0.0056 ± 0.0006	0.56 ± 0.06
Hajbabaei et al., 2013 (Bus) (12)	1	6.0 ± 1.2 g/mile	1868 ± 130 g/mile	0.0088 ± 0.0018	0.87 ± 0.18
Olofsson et al., 2014 (Bus) (19)	1	0.32 ± 0.07 g/km	967 ± 14 g/km	0.0009 ± 0.0002	0.09 ± 0.02
Clark et al., 2017 (Bus) (14)	3			0.0078 ± 0.0027	0.78 ± 0.27
Thiruvengadam et al., 2016 (Truck) (20)	4	3 ± 2 g/mile	2250 g/mile	0.0035 ± 0.0025	0.35 ± 0.25
Karavalakis et al., 2016 (Truck) (13)	2	5 ± 3 g/mile	2153 ± 65 g/mile	0.006 ± 0.004	0.6 ± 0.4
Grigoratos et al., 2016 (Truck) (21)	1	0.22 g/km	529.4 g/km	0.0011	0.11
Clark et al., 2017 (Truck) (20)	11			0.003 ± 0.003	0.3 ± 0.3
Average (Bus)[c]	16			0.004 ± 0.005	0.4 ± 0.5
Average (Truck)[c]	17			0.003 ± 0.003	0.3 ± 0.3
Average[c]				0.004 ± 0.004	0.4 ± 0.4
**Crankcase emissions from stoichiometric engines with three-way catalyst**					
Clark et al., 2017 (14)	11				1.0 ± 0.7
**Dual fuel diesel and natural gas engines with oxidation catalyst**					
Olofsson et al., 2014 (19)	1	7.6 ± 1.1 g/km	564 ± 67 g/km	0.037 ± 0.007	3.7 ± 0.7
Stettler et al., 2017 (22)	4	27 ± 16 g/km	735 ± 16 g/km	0.10 ± 0.06	10 ± 6
Average[c]				0.09 ± 0.06	9 ± 6
**High-pressure direct injection engines**					
Thiruvengadam et al., 2016 (20)	1	2.705 ± 0.018 g/mile	2150 g/mile	0.00346 ± 0.00006	0.345 ± 0.006
Clark et al., 2017 (14)	4			0.008 ± 0.004	0.8 ± 0.4
Average[c]				0.0078 ± 0.0046	0.78 ± 0.46

**Dynamic venting emissions from high-pressure direct injection engines**				
Clark et al., 2017 (14)	4		0.01 ± 0.01	1 ± 1

[a] Standard error is shown in the table; [b] Converted from total hydrocarbon (THC) emission factors assuming that 90% of THC was CH^4; [c] Sample-size weighted average; [d] Urban results from Yue et al. were used here to be consistent with other studies.

Supplementary Table 3. Instrumentation of the mobile platform.

	Precision	Frequency	Model and manufacturer	Measurement principle
**NH_3**	0.2 ppbv	10 Hz	Zondlo group(23)	Open-path, quantum cascade-laser-based sensor; Scanning a single NH_3 absorption feature at 9.06 µm to minimize interference from other gases; Wavelength modulation spectroscopy (WMS) is used to improve the performance.
**N_2O/ CO**	0.07 ppbv for N_2O, 0.2 ppbv for CO	10 Hz	Zondlo group(24)	Open-path, quantum cascade-laser-based sensor; Scanning single N_2O and single CO absorption features simultaneously at 4.54 µm to minimize interference; Wavelength modulation spectroscopy (WMS) is used to improve the performance.
**CH_4**	2 ppbv	10 Hz	LICOR-7700	Open-path, near-infrared tunable-laser based sensor; Scanning a single CH_4 absorption feature at 1.65 µm to minimize interference from other gases; Wavelength modulation spectroscopy (WMS) is used to improve the performance.
**H_2O/CO_2**	<1% for H_2O, 0.1 ppmv for CO_2	10 Hz	LICOR-7500	Non-dispersive infrared (NDIR) spectrometer.
**Pressure, temperature, and wind**	±0.5 hPa at 0 - 30 °C<br>±0.3 °C<br>±0.3 m/s or ±3%, whichever is greater	1 Hz	Vaisala WXT520	Wind speed and wind direction are measured by ultrasonic transducers; Pressure and temperature are measured by capacitive silicon sensor and capacitive ceramic sensor.
**Location**	10 meters	10 Hz	GlobalSat EM-406a	GPS

Supplementary Table 4. Engine characteristics and price of NG buses, NG trucks and diesel trucks in China.[a]

Vehicle Manufacturer	Engine manufacturer	Engine model	Engine type	Displacement	Power rating (hp)	Emission standard	Price (MSRP, CNY)
			**Natural buses**[b]				
Huanghai Bus Co. Ltd	Yuchai Machinery Co. Ld.	YC6G260N-50	Lean-burn with oxidation catalyst	7.8 L	260 (194) kW) @ 2300 rpm	China V	N/A
Huanghai Bus Co. Ltd	Yuchai Machinery Co. Ld.	YC6G260N-40	Lean-burn with oxidation catalyst	7.8 L	260 (194kW) @ 2300 rpm	China IV	N/A
Zhengzhou Yutong Bus Co.,Ltd.	Weichai Holding Group Co.,Ltd.	WP6NG210E40	Lean-burn with oxidation catalyst	6.75 L	210 (157 kW) @ 1300 - 1500	China IV	N/A
			**Natural trucks**				
Dongfeng Special Commercial Vehicle Co., Ltd.	Yuchai Machinery Co. Ltd.	YC6L280N-52	Lean-burn with oxidation catalyst	8.4 L	220 (164 kW) @ 2200 RPM	China V	343800
China National Heavy Duty Truck Group Co.,Ltd.	China National Heavy Duty Truck Group Co.,Ltd.	HW16709XSTC	Lean-burn	11.596 L	380 (283kW) @ 2000 RPM	China V	346700
Weichai Holding Group Co.,Ltd.	Weichai Holding Group Co.,Ltd.	WP10NG336E50	Lean-burn with oxidation catalyst	9.726 L	336 (250 kW) @ 2200 RPM	China IV	350000
			**Dongfeng Special Commercial Vehicle Co., Ltd.**				
Dongfeng Special Commercial Vehicle Co., Ltd.	Dongfeng Renault Co., Ltd.	dCi350-51	Diesel	11 L	350 (261 kW) @ 2200 RPM	China V	289500
China National Heavy Duty Truck Group Co.,Ltd.	China National Heavy Duty Truck Group Co.,Ltd.	D10.38-50	Diesel	9.726L	380 (284 kW) @ 2000 RPM	China V	290000
Weichai Holding Group Co.,Ltd.	Weichai Holding Group Co.,Ltd.	WP10.336E40	Diesel	9.726 L	336 (250 kW) @ 1900 RPM	China IV	293400

[a] Information obtained from http://buses.cn and http://www.360che.com, in 2018/11.
[b] These engines were also equipped on trucks.

Supplementary Table 5. Cold/hot start CH_4 emission ratios. Cold-start tests carried out by Olofsson et al. (2014) and Stettler et al. (2018) were at −2°C and 0°C respectively.

	**Start condition**	**CH_4 g/km**	**CO_2 g/km**	**Fuel-specific emission factor (%)**	**Cold/hot start emission ratio**
Olofsson et al. (2014) Lean-mix with OC and SCR (20)	hot	0.5	660	0.2	2.69
	cold	1.7	843	0.5	
Stettler et al. (2018) Dual-fuel SCR (9.2 L) (22)	hot	9.2	705	3.6	1.37
	cold	12.8	714	4.9	
Stettler et al. (2018) Dual-fuel OC + SCR (9.2 L) (22)	hot	8.8	753	3.2	1.66
	cold	14.4	744	5.3	
Stettler et al. (2018) Dual-fuel OC + SCR (12.0 L) (22)	hot	29.7	730	11.2	1.08
	cold	33.0	751	12.1	

Supplementary Table 6. Well-to-wheels emissions of gasoline, diesel, and natural gas vehicles in China.

Source	Well-to-wheels GHG emissions (g CO_{2eq})/MJ	Well-to-pump CH_4 leakage (%)	Note
**Gasoline**			
Ou et al., 2010 (25)	98.86		
Huo et al., 2012 (26)	98		
Ding et al., 2013 (27)	98		
Value used in this study	98.00		
**Diesel**			
Ou et al., 2010 (25)	102.4		
Song et al., 2017 (28)	92.36		
Value used in this study	97.38 ± 7.10		
**Compressed natural gas without vehicle CH_4 emissions**			
Yan et al., 2009 (29)	74 - 80	N/A	
Huo et al., 2012 (26)	84.4 ± 5.5	1.93% + 0.0007%×pipeline distance (km)	1100 km pipeline transport for NG
Ou et al., 2013 (30)	73.2	N/A	500 km pipeline transport for NG
Value used in this study	83 ± 6	1.65% ± 1.05%	1100 km pipeline transport for NG with standard error from Huo et al. to represent uncertainty in NG transport distance
**Liquified natural gas without vehicle CH_4 emissions**			
Ou et al., 2013, imported NG (30)	75.7	N/A	6700 km pipeline transport from oversea source region, followed by 100 km truck transport
Ou et al., 2013 (30)	77.5	N/A	100 km truck transport
Ou et al., 2013 (30)	78.5	N/A	100 km pipeline transport
Song et al., 2017 (28)	77.48	0.60%	
Value used in this study	84 ± 6	1.65% ± 1.05%	1100 km pipeline transport for NG with standard error from Huo et al. to represent uncertainty in NG transport distance
**Natural gas with vehicle CH_4 emissions**			
This study, current, light-duty vehicles	95 ± 8	1.65% ± 1.05%	
This study, current, heavy-duty buses	103 [-9, 10]	1.65% ± 1.05%	
This study, current, heavy-duty trucks	103 [-11, +10]	1.65% ± 1.05%	
This study, SM with TWC	92 ± 8	1.65% ± 1.05%	Crankcase emissions are included
This study, HPDI	94 ± 9	1.65% ± 1.05%	Dynamic venting emissions are included
This study, China VI	88 ± 6	1.65% ± 1.05%	

Supplementary Table 7. Fuel consumption used in this study. Fuel consumption for both diesel and natural gas vehicles purchased after 2021 is lowered by 15% assuming the China Stage 3 Fuel Consumption Limits will be implemented stringently (35).

**Source**	**Vehicle type**	**Fuel consumption (MJ/km)**
Huo et al., 2012 (26)	Gasoline light-duty vehicle	2.55
Huo et al., 2012 (26)	Light-duty NGV	2.73
Zhang et al., 2014 (31)	Diesel bus	11.8 ± 2.6
Zhang et al., 2014 (31)	Natural gas bus[b]	14.7 ± 1.0
Song et al., 2017 [a, (28)]	Diesel truck	15.9 ± 0.8
Song et al., 2017 [a, (28)]	Natural gas truck[b]	17.3 ± 0.9

[a] Sub-category vehicle population weighted average from Song et al., 2017 (28).
[b] For HPDI engines, fuel consumptions listed here were scaled by 0.95 to reflect that HPDI engines have higher efficiency compared to LB and SM engines (19).

Supplementary Table 8. Annual mileage travelled (AMT, $\times 10^4$ km) for the four categories of NGVs. Taxis in China are required to retire after 8-year operation.

**Age**	**Light-duty vehicle** [a]	**Taxi** [a]	**Heavy-duty truck** [b]	**Heavy-duty bus** [a]
1	2.03	10	15	6.3
2	2.02	9.8	14.7	6.2
3	2	9.5	14.5	6.1
4	1.94	9.2	14.3	6
5	1.88	9	13.8	5.8
6	1.82	8.7	13.4	5.6
7	1.76	8.5	13.4	5.6
8	1.69	8	13.4	5.6
9	1.65		13.4	5.6
10	1.61		13.4	5.6
11	1.55		10.7	4.5
12	1.51		10.7	4.5
13	1.47		8.3	3.5
14	1.4		8.3	3.5
15	1.34		8.3	3.5

[a] Taxies are excluded, He et al., 2010(32); [b] Sub-category vehicle population weighted average from Song et al., 2017(28).

Supplementary Table 9. Projected vehicle population of light-duty NGV, heavy-duty NG bus, heavy-duty NG. The penetration rates of heavy-duty NG bus and heavy-duty NG truck for the medium-emission scenario are adopted from Wu et al., 2017.

Year	Total NGV	Light-duty NGVs without Taxi	Heavy-duty NG bus	Heavy-duty NG truck	NG taxi
		**High-emission scenario**			
2018	7000000	5483333	310000	406667	800000
2019	8500000	6781667	330000	488333	900000
2020	10000000	8080000	350000	570000	1000000
2021	10188000	8080000	375000	733000	1000000
2022	10376000	8080000	400000	896000	1000000
2023	10564000	8080000	425000	1059000	1000000
2024	10752000	8080000	450000	1222000	1000000
2025	10940000	8080000	475000	1385000	1000000
2026	11128000	8080000	500000	1548000	1000000
2027	11316000	8080000	525000	1711000	1000000
2028	11504000	8080000	550000	1874000	1000000
2029	11692000	8080000	575000	2037000	1000000
2030	11880000	8080000	600000	2200000	1000000
		**Medium-emission scenario**			
2018	7000000	5483333	310000	406667	800000
2019	8500000	6781667	330000	488333	900000
2020	10000000	8080000	350000	570000	1000000
2021	10021191	7969191	363000	689000	1000000
2022	10049800	7865800	376000	808000	1000000
2023	10037613	7721613	389000	927000	1000000
2024	10055242	7607242	402000	1046000	1000000
2025	9905946	7325946	415000	1165000	1000000
2026	9510875	6798875	428000	1284000	1000000
2027	8573368	5729368	441000	1403000	1000000
2028	7570265	4594265	454000	1522000	1000000
2029	7242315	4134315	467000	1641000	1000000
2030	7112558	3872558	480000	1760000	1000000
		**Low-emission scenario**			
2018	7000000	5581333	310000	406667	702000
2019	8500000	6979667	330000	488333	702000
2020	10000000	8378000	350000	570000	702000
2021	9965191	8267191	351000	645000	702000
2022	9937800	8163800	352000	720000	702000
2023	9869613	8019613	353000	795000	702000
2024	9831242	7905242	354000	870000	702000
2025	9625946	7623946	355000	945000	702000
2026	9174875	7096875	356000	1020000	702000
2027	8181368	6027368	357000	1095000	702000
2028	7122265	4892265	358000	1170000	702000
2029	6738315	4432315	359000	1245000	702000
2030	6552558	4170558	360000	1320000	702000

Supplementary Table 10. CH_4/CO_2 ER uncertainty for daily fleet-wide sample. Mean and standard deviation are sample-size weighted (seconds directly after vehicles). CH_4 threshold choices lead to a large uncertainty compare to sample variability and dominate the final uncertainty.

	6/10/2014	6/11/2014	6/12/2014	Mean	Uncertainty (Standard deviation)
**Bus sample size (seconds directly after buses)**	1993	3015	617		
**Bus sample size (number of buses)**	26	34	13		
**Taxi sample size (seconds directly after taxis)**	863	1953	710		
**Taxi sample size (number of taxis)**	20	27	16		
			**Default criteria**		
**Bus emission ratio (ppmv/ppmv)**	0.0287	0.0294	0.0273	0.0289	0.0008
**Taxi emission ratio (ppmv/ppmv)**	0.0211	0.0159	0.0159	0.0172	0.002
			**No CH_4 threshold**		
**Bus emission ratio (ppmv/ppmv)**	0.0249	0.0241	0.0217	0.0240	0.0012
**Taxi emission ratio (ppmv/ppmv)**	0.0145	0.0115	0.0103	0.0121	0.002
			**0.4 ppmv CH_4 threshold**		
**Bus emission ratio (ppmv/ppmv)**	0.0318	0.0348	0.0327	0.0335	0.0014
**Taxi emission ratio (ppmv/ppmv)**	0.0283	0.0202	0.0203	0.0223	0.004
			**Plume identification uncertainty ($(ER_{0.4\ ppmv\ threshold} – ER_{no\ threshold})/2$)**		
**Bus emission ratio (ppmv/ppmv)**	0.0034	0.0053	0.0056		
**Taxi emission ratio (ppmv/ppmv)**	0.0069	0.0043	0.0050		

Supplementary Table 11. Relative uncertainty (standard error) of the parameters (vehicle population (VP), annual mileage traveled (AMT), fuel consumption (FC), and emission ratio (ER)) used in the compilation of CH_4 emission inventory. Data from official sources or literature were given 10% relative uncertainty unless the uncertainty was provided or can be calculated. Data from unofficial sources were given 20% relative uncertainty. Data derived by linear interpolation were given 30% relative uncertainty.

Year	VP[a] of LDNGV[b]	VP of NG taxi	VP of HDNGT[c]	VP of HDNGB[d]	AMT of LDNGV[b]	AMT of NG taxi	AMT of HDNGT	AMT of HDNGB	FC[e] of LDNGV[f]	FC of HDNGB	FC of HDNGB	ER[g] of LDNGV[f]	ER of HDNGB	ER of HDNGT[ss]
2000	20%	30%	-	20%	10%	10%	10%	10%	5%	5%	7%	[-36, +47] %	[-25, +31] %	[−53, +31] %
2001	20%	30%	-	20%	10%	10%	10%	10%	5%	5%	7%	[-36, +47] %	[-25, +31] %	[−53, +31] %
2002	20%	30%	-	20%	10%	10%	10%	10%	5%	5%	7%	[-36, +47] %	[-25, +31] %	[−53, +31] %
2003	20%	30%	-	20%	10%	10%	10%	10%	5%	5%	7%	[-36, +47] %	[-25, +31] %	[−53, +31] %
2004	20%	30%	-	20%	10%	10%	10%	10%	5%	5%	7%	[-36, +47] %	[-25, +31] %	[−53, +31] %
2005	20%	30%	-	20%	10%	10%	10%	10%	5%	5%	7%	[-36, +47] %	[-25, +31] %	[−53, +31] %
2006	20%	30%	-	20%	10%	10%	10%	10%	5%	5%	7%	[-36, +47] %	[-25, +31] %	[−53, +31] %
2007	20%	30%	20%	20%	10%	10%	10%	10%	5%	5%	7%	[-36, +47] %	[-25, +31] %	[−53, +31] %
2008	20%	30%	20%	20%	10%	10%	10%	10%	5%	5%	7%	[-36, +47] %	[-25, +31] %	[−53, +31] %
2009	20%	30%	20%	20%	10%	10%	10%	10%	5%	5%	7%	[-36, +47] %	[-25, +31] %	[−53, +31] %
2010	20%	10%	20%	20%	10%	10%	10%	10%	5%	5%	7%	[-36, +47] %	[-25, +31] %	[−53, +31] %
2011	20%	10%	20%	20%	10%	10%	10%	10%	5%	5%	7%	[-36, +47] %	[-25, +31] %	[−53, +31] %
2012	20%	10%	20%	20%	10%	10%	10%	10%	5%	5%	7%	[-36, +47] %	[-25, +31] %	[−53, +31] %
2013	20%	10%	30%	30%	10%	10%	10%	10%	5%	5%	7%	[-36, +47] %	[-25, +31] %	[−53, +31] %
2014	20%	10%	30%	30%	10%	10%	10%	10%	5%	5%	7%	[-36, +47] %	[-25, +31] %	[−53, +31] %
2015	20%	10%	20%	20%	10%	10%	10%	10%	5%	5%	7%	[-36, +47] %	[-25, +31] %	[−53, +31] %
2016	20%	20%	30%	30%	10%	10%	10%	10%	5%	5%	7%	[-36, +47] %	[-25, +31] %	[−53, +31] %
2017	20%	20%	20%	20%	10%	10%	10%	10%	5%	5%	7%	[-36, +47] %	[-25, +31] %	[−53, +31] %

[a] VP: Vehicle population; [b] LDNGV: light-duty NGV; [c] HDNGT: heavy-duty NG truck; [d]: HDNGB: heavy-duty NG bus; [e] FE: fuel economy; [f] The same value is used for NG taxi; [g] ER: emission ratio. [h] NG trucks may operate on highway more often than NG buses, resulting in lower CH_4 emissions. Hence, we assigned a larger standard error for the lower bound of EF of NG trucks that is equivalent to the lower bound of the previously reported CH_4 EF of LB engines with OC.

Supplementary Table 12. Values of parameters used in PUFFER.

Parameter, symbol (units)	Value	Parameter, symbol (units)	Value
Canyon length, L (m)	600	CO_2 emission factor	
Canyon width, B (m)	20	(idle (kg/s), velocity (kg m^{-1}), acceleration (kg $m^{-1}s^{-1}$)	
Canyon height, H (m)	20	NGV	(0.045, 0.15, 0.45)
Vehicle dimension		Mobile lab	(0.009, 0.03, 0.09)
(length (m), width (m), height(m))		Vehicle 1-4	(0.009, 0.03, 0.09)
NGV	(9, 2.5, 2.5)	CH4 emission factor	
Mobile lab	(3, 1.67, 1.8)	(idle (g/s), velocity (g m-1), acceleration (g $m^{-1}s^{-1}$)	
Vehicle 1-4	(2.5, 1.5, 1.8)	NGV	(2.5, 5, 7.5)
Wind speed, u_H	0	Mobile lab	(0, 0, 0)
Wind speed, v_H	-4	Vehicle 1-4	(0, 0, 0)
Turbulence parameter, α_1	0.25	Time step	0.05
Turbulence parameter, α_2	0.1	No. of time steps	1200

Supplementary Table 13. Estimated mean $\Delta CH_4/\Delta CO_2$ ratios using CO_2 and CH_4 concentrations simulated by PUFFER and random walk PUFFER. The results from random walk PUFFER are the mean values from 50 simulations, and values in parentheses are the standard deviations.

	**PUFFER**		**Random walk PUFFER**		**True value**
	Slope of orthogonal regression	Quotient	Slope of orthogonal regression	Quotient	
Mean $\Delta CH_4/\Delta CO_2$ (ppmv/ppmv)	0.0282	0.0310	0.0318 (0.0007)	0.0315 (0.0005)	0.0318

Supplementary Discussion

The plume chasing method described in the manuscript assumes that CH_4 and CO_2 are co-emitted from the sources of interest and are dispersed in the same way. To make sure this assumption is valid, we only used observations when the mobile was directly following NGVs. An example is provided in “SI_Method_Demo.mp4”, and the observations are also shown in Supplementary Figure 5. As shown in “SI_Method_Demo.mp4”, there were still vehicles in other lanes and occasionally appeared between the NGVs and the mobile laboratory. Since most of the vehicles were not powered by NG, emissions from these vehicles would contribute to the observed ΔCO_2 but not ΔCH_4, which could potentially lower estimated emission ratios. To reduce such interference, we implemented a threshold for correlation between ΔCO_2 and ΔCH_4 using R^2. We excluded observations when R^2 was lower than 0.5 within the ±1 second window. The assumption behind this criterion is that high-frequency variations (10 Hz) of CO_2 and CH_4 concentrations are caused by turbulent movements of the plumes, and these changes should be correlated if CO_2 and CH_4 plumes are emitted from the same source. In addition, the slopes from orthogonal regressions between ΔCH_4 and ΔCO_2 instead of their quotients were used to derive emission ratios, which could help remove the ΔCO_2 offsets caused by emissions from other vehicles. To investigate the effectiveness of our method, we used a Gaussian puff model to simulate on-road CO_2 and CH_4 concentrations measured by our mobile laboratory.

The Gaussian puff model, PUFFER, was developed to model the dispersion of vehicular pollutants in the urban street canyon environment proposed by Hargreaves and Baker[36, 37]. We conducted a 60-second simulation with a time step (Δt) of 0.05 second (1200 steps) for a street canyon with dimensions of 600 × 20 × 20 m with two contraflowing lanes of traffic. The speed and location of each vehicle were updated at each time step according to predetermined acceleration time series. Supplementary Figure 6 shows the trajectories of the vehicles.

Each vehicle emits a small “puff” of exhaust containing CO_2 and CH_4, and each puff maintains a 3D Gaussian distribution characterized by the three dispersion parameters, σ_x, σ_y, and σ_z. The transport and dispersion of the puffs is simulated by two separate processes: the movement of the vehicles and the above canyon wind. The along-canyon component of a puff’s velocity caused by the wake of a vehicle is modeled as

$$u_w = u_v (s/h)^{-3/4} \varsigma \exp[-(\varsigma^2 + \eta^2)/8], \; s > 0 \tag{1}$$

with

$$\begin{cases} \varsigma = z_p / h_w \\ \eta = (y_p - y_v) / h_w \end{cases} \tag{2}$$

where u_v is the vehicle’s along-canyon speed, s is the distance of the puff from the front of the vehicle, $h_w = h(s/h)^{1/4}$ is the wake height, h is the vehicle height, and (x_v, y_v, z_v) and

(x_p, y_p, z_p) are the coordinates of the centers of the rear of the vehicle and the puff. The above canyon wind, when not aligned along the street, is responsible for the vortex patterns of airflow in the canyon. The flow patterns are simulated as

$$\begin{cases} u_a = u_H \log[(z+z_0)/z_0]/\log[(H+z_0)/z_0] \\ v_a = v_H \log[e^{kz'}(1+kz') - \gamma e^{-kz'}(1-kz')]\sin ky/(1-\gamma) \\ w_a = -v_H kz'[e^{kz'} - \gamma e^{-kz'}]\cos ky/(1-\gamma) \end{cases} \quad (3)$$

where $k = \pi / B$, $\gamma = \exp(-2kH)$, $z' = z - H$, and u_H and v_H are the along and cross canyon components of the above canyon wind, and z_0 is a parameter analogous to the surface roughness. In our simulation, it is assumed $u_H = 0$ and $v_H = -4$ m/s and Supplementary Figure 7 shows the flow pattern along the cross section of the street canyon. Each puff is moved according to the sum of speeds listed in Eq. (1) and (3).

The dispersion parameters of the puff describe the extent to which the puff has expanded. The initial rapid expansion due to the relatively large turbulence close to the rear of the vehicle is calculated as

$$\begin{cases} \sigma_x^0 = \frac{1}{4}(h+b) \\ \sigma_y^0 = \frac{1}{2}b \\ \sigma_z^0 = \frac{1}{2}h \end{cases} \quad (4)$$

where b is the width of the vehicle. After the short initial burst of mixing, the puffs expand according to

$$\sigma_i^{r+1} = \sigma_i^r + \overline{(u_i'^2)}^{1/2} \Delta t \quad (5)$$

where $\overline{(u_i'^2)}^{1/2}$ is the *i*th component of the turbulence in the canyon, which is estimated as

$$\overline{(u_i'^2)}^{1/2} = \alpha_1[\alpha_2 u_i^2 + (1-\alpha_2)(u_j^2 + u_k^2)]^{1/2} \quad (6)$$

where α_1 is a parameter which relates the level of turbulence to the local wind velocity, α_2 is a parameter which quantifies the level of anisotropy in the turbulence.

The concentrations of CH_4 and CO_2 observed by the mobile laboratory can be calculated by

$$c(x_m, y_m, z_m, t) = \sum_{p=1}^{N_p} \left(\frac{Q\Delta t}{(2\pi)^{3/2} \sigma_{x,p} \sigma_{y,p} \sigma_{z,p}} \right) X(x_m) Y(y_m) Z(z_m) \tag{7}$$

with

$$\begin{cases} X(x_r) = \exp\left[-\frac{1}{2} \left(\frac{x_m - x_p}{\sigma_{x,p}} \right)^2 \right] \\ Y(y_r) = \exp\left[-\frac{1}{2} \left(\frac{y_m - y_p}{\sigma_{y,p}} \right)^2 \right] + \exp\left[-\frac{1}{2} \left(\frac{y_m + y_p}{\sigma_{y,p}} \right)^2 \right] + \exp\left[-\frac{1}{2} \left(\frac{2B - y_m - y_p}{\sigma_{y,p}} \right)^2 \right] \\ Z(z_r) = \exp\left[-\frac{1}{2} \left(\frac{z_m - z_p}{\sigma_{z,p}} \right)^2 \right] + \exp\left[-\frac{1}{2} \left(\frac{z_m + z_p}{\sigma_{z,p}} \right)^2 \right] \end{cases} \tag{8}$$

where (x_m, y_m, z_m) is the location of the sensors and Q (g/s) represents the emission rates of CO_2 or CH_4 when the puff is emitted. All the sensors are assumed to be collocated. x_m and y_m are the same as the center of the mobile laboratory, whereas z_m is 0.33 m above the roof of the mobile laboratory (2.0 m above the ground). The emission rate is modeled simply as

$$Q = q_1 + u_v q_2 + a_v q_3 \tag{9}$$

where q_1 is the idling emission rate, q_2 and q_3 are the coefficients for velocity and acceleration-dependent emissions. Because CH_4 emissions from conventional gasoline or diesel vehicles are 10-200 times lower than NGVs, CH_4 emissions from the mobile laboratory and Vehicle 1-4 are ignored. Parameters used in the Gaussian puff model are listed in Supplementary Table 12. Since there could be more leakage of CH_4 from the NGVs, idling emissions have a larger contribution for CH_4 emissions compared to CO_2 emissions. Acceleration related emissions are also lower for CH_4 compared to CO_2 to demonstrate the effects of changing emission ratios. The bottom two panels of Supplementary Figure 6 show CO_2 and CH_4 emissions from the vehicles.

Supplementary Figure 8 shows the simulated CO_2 and CH_4 observations as well as the true and estimated emission ratios. As discussed by Baker and Hargreaves, the Gaussian puff model discussed above describes the ensemble average behavior of the puff, and the inherent time scale of the PUFFER model is on the order of a few seconds[37]. Therefore, it is expected that the results from PUFFER lack the high-frequency variations (>1 Hz) found in our observations. Missing the high-frequency variations worsens the performance of our method. The slopes of orthogonal regression deviate from the true ΔCH_4: ΔCO_2 ratios when the observations are influenced by other vehicles, and the R^2 criterion failed to remove the interferences. Since the high-frequency variations are essential for the correlation criterion and the orthogonal regression method, we introduced a random walk mechanism that adds high-frequency variations to the simulated time

series while preserving the statistical properties of PUFFER. At each time step, the center of the puff moves along x-, y-, and z-axis with step sizes ($\delta x,\ \delta y,\ \delta \mathrm{z}$) that vary according to normal distributions. The initial normal distributions are

$$\begin{cases} \delta x_0 \sim N(u_0 \Delta t, \dfrac{\sigma_x^0}{\sqrt{2}}) \\ \delta y_0 \sim N(v_0 \Delta t, \dfrac{\sigma_y^0}{\sqrt{2}}) \\ \delta z_0 \sim N(w_0 \Delta t, \dfrac{\sigma_z^0}{\sqrt{2}}) \end{cases} \tag{10}$$

After the initial expansion, the step sizes vary following normal distributions

$$\begin{cases} \delta x_i \sim N(u_i \Delta t, \dfrac{\overline{(u_{i-1}'^2)}^{1/2} \Delta t}{\sqrt{2}}) \\ \delta y_i \sim N(v_i \Delta t, \dfrac{\overline{(v_{i-1}'^2)}^{1/2} \Delta t}{\sqrt{2}}) \qquad ,\ i > 0 \\ \delta z_{10i+j} \sim N(w_i \Delta t, \dfrac{\overline{(w_{i-1}'^2)}^{1/2} \Delta t}{\sqrt{2}}) \end{cases} \tag{11}$$

The expected puff center and its standard deviation can be calculated as

$$\begin{cases} \overline{x_{pr}}(t) = \sum_{i=0}^{t} \delta x_i = x_p(t) + \sum_{i=0}^{t} u_i \Delta t = x_p(t) \\ \overline{y_{pr}}(t) = \sum_{i=0}^{t} \delta y_i = y_p(t) + \sum_{i=0}^{t} v_i \Delta t = v_p(t) \\ \overline{x_{pr}}(t) = \sum_{i=0}^{t} \delta z_i = z_p(t) + \sum_{i=0}^{t} w_i \Delta t = z_p(t) \end{cases} \tag{12}$$

$$\begin{cases} \sigma_{xr}(t) = \dfrac{1}{\sqrt{2}} \left[\sigma_x^0 + \sum_{i=0}^{t} \overline{(u_i'^2)}^{1/2} \Delta t \right] = \dfrac{\sigma_x^t}{\sqrt{2}} \\ \sigma_{yr}(t) = \dfrac{1}{\sqrt{2}} \left[\sigma_y^0 + \sum_{i=0}^{t} \overline{(v_i'^2)}^{1/2} \Delta t \right] = \dfrac{\sigma_y^t}{\sqrt{2}} \\ \sigma_{zr}(t) = \dfrac{1}{\sqrt{2}} \left[\sigma_z^0 + \sum_{i=0}^{t} \overline{(w_i'^2)}^{1/2} \Delta t \right] = \dfrac{\sigma_z^t}{\sqrt{2}} \end{cases} \tag{13}$$

It should be noted that σ_{xr}, σ_{yr}, and σ_{zr} describe the spread of puff center instead of the spread of the puff itself. The spatial distributions of CH_4 and CO_2 are still modeled with Gaussian distribution, but with the dispersion parameters reduced to $1/\sqrt{2}$ of their original values. It can be shown that the ensemble mean of the random walk PUFFER is identical to the original model. For example, considering *x* direction alone, the expected concentration of CH_4 or CO_2 can be expressed as

$$\begin{aligned}
\overline{c(x_m, x_p, t)} &= \int_{-\infty}^{\infty} c(x_m, x_{pr}, t)\, p(x_{pr} \mid x_p, t)\, dx_{pr} \\
&= \int_{-\infty}^{\infty} \frac{Q\Delta t}{\sqrt{2\pi}\sigma_x^t / \sqrt{2}} \exp\left[-\frac{1}{2}\frac{(x_m - x_{pr})^2}{(\sigma_x^t)^2/2}\right] \frac{1}{\sqrt{2\pi}\sigma_x^t/\sqrt{2}} \exp\left[-\frac{1}{2}\frac{(x_p - x_{pr})^2}{(\sigma_x^t)^2/2}\right] dx_{pr} \\
&= \frac{Q\Delta t}{\pi(\sigma_x^t)^2} \int_{-\infty}^{\infty} \exp\left\{-\frac{1}{(\sigma_x^t)^2}\left[(x_m - x_{pr})^2 + (x_p - x_{pr})^2\right]\right\} dx_{pr} \\
&= \frac{Q\Delta t}{2\pi(\sigma_x^t)^2} \int_{-\infty}^{\infty} \exp\left\{-\frac{1}{(\sigma_x^t)^2}\left[\frac{1}{2}(x_m - x_p)^2 + \frac{1}{2}(2x_{pr} - x_p - x_m)^2\right]\right\} d(2x_{pr}) \\
&= \frac{Q\Delta t}{\sqrt{2\pi}\sigma_x^t} \exp\left[-\frac{1}{2}\frac{(x_m - x_p)^2}{(\sigma_x^t)^2}\right]
\end{aligned} \tag{14}$$

Eq. (14) is identical to Eq. (7) when only *x*-direction is considered. Supplementary Figure 9 shows the ensemble mean of 100 profiles at t=30 second of a puff emitted at t=5 second simulated using random walk PUFFER. And the ensemble mean profiles are indeed consistent with the original Gaussian puff model.

Supplementary Figure 10 (a) and (b) show an example of simulated CO_2 and CH_4 concentrations. Introducing randomness to PUFFER added the necessary high-frequency variations. The true ΔCH_4: ΔCO_2 ratios (blue), and the ΔCH_4: ΔCO_2 ratios derived as the slopes of orthogonal regression (orange) and as the quotients (green) are shown in Supplementary Figure 9 (c). The slopes of orthogonal regression are less impacted by emissions from other vehicles compared to the results from the original PUFFER. Supplementary Figure 9 (d) shows the determination coefficients (R^2), and R^2 are lower as expected when the emissions from nearby vehicles contribute to CO_2 enhancements. Setting the threshold of R^2 to be 0.5 can remove a large fraction of invalid observations. Sensitivity tests of the threshold of R^2 is discussed in the Method section in the manuscript. Since the results from random walk PUFFER are not deterministic, 50 simulations were conducted, and the statistics of the mean slopes and quotients filtered by R^2 are listed in Supplementary Table 13. Our method shows no statistically significant difference from the true value when high-frequency variations are considered.

Random walk PUFFER by no means reflects the true atmospheric turbulence in urban environment, which is still a frontier research topic and is beyond the scope of this study[38].

However, random walk PUFFER allows us to introduce high-frequency transient changes in CH_4 and CO_2 concentrations and is, thus, a powerful tool to evaluate our method. Our investigation above supported our assumption that high-frequency variations (10 Hz) of CO_2 and CH_4 concentrations should be correlated if CO_2 and CH_4 plumes are emitted from the same source and go through the same atmospheric transport. Our results also highlight the importance of capturing high-frequency variations. When high-frequency variations are present, which was usually the case for busy roads when our measurements were influenced by other vehicles, our method can significantly reduce the interference.

Supplementary References

1. Ministry of Transport of the People's Republic of China (2010 - 2014) *China Transport Statistical Yearbook*.
2. Xie S, *et al.* (2011) Real-world emission characteristics of natural gas-gasoline bi-fuel vehicles. *Acta Scientiae Circumstantiae* 31(11):2347-2353.
3. Hu N, *et al.* (2018) Large methane emissions from natural gas vehicles in Chinese cities. *Atmospheric Environment*.
4. Karavalakis G, Durbin TD, Villela M, & Miller JW (2012) Air pollutant emissions of light-duty vehicles operating on various natural gas compositions. *Journal of Natural Gas Science and Engineering* 4:8-16.
5. Bielaczyc P, Woodburn J, & Szczotka A (2014) An assessment of regulated emissions and CO2 emissions from a European light-duty CNG-fueled vehicle in the context of Euro 6 emissions regulations. *Applied Energy* 117:134-141.
6. Burnham A, Wang M, & Wu Y (2006) Development and applications of GREET 2.7--The Transportation Vehicle-CycleModel. (Argonne National Lab.(ANL), Argonne, IL (United States)).
7. Lima G, *et al.* (2010) Detection of greenhouse gases emitted by engines powered by natural gas. *International journal of environmental studies* 67(6):837-849.
8. Nilrit S, Sampanpanish P, & Bualert S (2013) EMISSION FACTORS OF CH^ sub 4^ AND CO^ sub 2^ EMITTED FROM VEHICLES. *American Journal of Environmental Sciences* 9(1):38.
9. Guo J, *et al.* (2014) On-road measurement of regulated pollutants from diesel and CNG buses with urea selective catalytic reduction systems. *Atmospheric environment* 99:1-9.
10. Yue T, *et al.* (2016) Gaseous emissions from compressed natural gas buses in urban road and highway tests in China. *Journal of Environmental Sciences* 48:193-199.
11. Yoon S, *et al.* (2013) Criteria pollutant and greenhouse gas emissions from CNG transit buses equipped with three-way catalysts compared to lean-burn engines and oxidation catalyst technologies. *Journal of the Air & Waste Management Association* 63(8):926-933.
12. Hajbabaei M, Karavalakis G, Johnson KC, Lee L, & Durbin TD (2013) Impact of natural gas fuel composition on criteria, toxic, and particle emissions from transit buses equipped with lean burn and stoichiometric engines. *Energy* 62:425-434.
13. Karavalakis G, *et al.* (2016) Regulated, greenhouse gas, and particulate emissions from lean-burn and stoichiometric natural gas heavy-duty vehicles on different fuel compositions. *Fuel* 175:146-156.
14. Clark NN, *et al.* (2017) Future methane emissions from the heavy-duty natural gas transportation sector for stasis, high, medium, and low scenarios in 2035. *Journal of the Air & Waste Management Association* 67(12):1328-1341.
15. Ministry of Environmental Protection of the People's Republic of China (2005) *Limits and Measurement Methods for Exhaust Pollutants from Compression Ignition and Gas Fuelled Positive Ignition Engines of Vehicles (III, IV, V)*

16. Ministry of Ecological Environment of the People's Republic of China (2018) *Limits and Measurement Methods for Emissions from diesel fuelled heavy-duty vehicles (China VI)*.

17. Hesterberg TW, Lapin CA, & Bunn WB (2008) A comparison of emissions from vehicles fueled with diesel or compressed natural gas. *Environmental science & technology* 42(17):6437-6445.
18. Nylund N-O & Koponen K (2012) Fuel and Tehnology Alternatives for Buses.
20. Olofsson M, Erlandsson L, & Willner K (2014) Enheanced Emission Performance and Fuel Efficiency for HD Methane Engines (Final Report). in *AVL MTC Report OMT 1032*.
19. Thiruvengadam A, *et al.* (2016) Unregulated greenhouse gas and ammonia emissions from current technology heavy-duty vehicles. *Journal of the Air & Waste Management Association* 66(11):1045-1060.
21. Grigoratos T, Fontaras G, Martini G, & Peletto C (2016) A study of regulated and green house gas emissions from a prototype heavy-duty compressed natural gas engine under transient and real life conditions. *Energy* 103:340-355.
22. Stettler ME, Midgley WJ, Swanson JJ, Cebon D, & Boies AM (2016) Greenhouse gas and noxious emissions from dual fuel diesel and natural gas heavy goods vehicles. *Environmental science & technology* 50(4):2018-2026.
23. Miller D, Sun K, Tao L, Khan M, & Zondlo M (2014) Open-path, quantum cascade-laser-based sensor for high-resolution atmospheric ammonia measurements. *Atmospheric Measurement Techniques* 7(1):81-93.
24. Tao L, Sun K, Khan MA, Miller DJ, & Zondlo MA (2012) Compact and portable open-path sensor for simultaneous measurements of atmospheric N 2 O and CO using a quantum cascade laser. *Optics express* 20(27):28106-28118.
25. Ou X, Zhang X, & Chang S (2010) Alternative fuel buses currently in use in China: life-cycle fossil energy use, GHG emissions and policy recommendations. *Energy Policy* 38(1):406-418.
26. Huo H, Zhang Q, Liu F, & He K (2013) Climate and environmental effects of electric vehicles versus compressed natural gas vehicles in China: A life-cycle analysis at provincial level. *Environmental science & technology* 47(3):1711-1718.
27. Ding Y, Han W, Chai Q, Yang S, & Shen W (2013) Coal-based synthetic natural gas (SNG): A solution to China's energy security and CO2 reduction? *Energy Policy* 55:445-453.
28. Song H, Ou X, Yuan J, Yu M, & Wang C (2017) Energy consumption and greenhouse gas emissions of diesel/LNG heavy-duty vehicle fleets in China based on a bottom-up model analysis. *Energy* 140:966-978.
29. Yan X & Crookes RJ (2009) Life cycle analysis of energy use and greenhouse gas emissions for road transportation fuels in China. *Renewable and Sustainable Energy Reviews* 13(9):2505-2514.
30. Ou X & Zhang X (2013) Life-cycle analyses of energy consumption and GHG emissions of natural gas-based alternative vehicle fuels in China. *Journal of Energy* 2013.
31. Zhang S, *et al.* (2014) Real-world fuel consumption and CO2 emissions of urban public buses in Beijing. *Applied Energy* 113:1645-1655.
32. HE L, *et al.* (2014) CH4 and N2O emission inventory for motor vehicles in China in 2010. *Research of Environmental Sciences* 27(1):28-35.
33. Bureau CS (2002 - 2018) China statistical yearbook. *National Bureau of Statistics, Beijing, China*.

34. Zhao Y, Nielsen CP, McElroy MB, Zhang L, & Zhang J (2012) CO emissions in China: uncertainties and implications of improved energy efficiency and emission control. *Atmospheric environment* 49:103-113.
35. General Administration of Quality Supervision. (ed Inspection and Quarantine of the People's Republic of China) (2018).
36. Hargreaves and Baker (1997) Gaussian puff model of an urban street canyon. *Journal of Wind Engineering and Industrial Aerodynamics* 69-71: 927-939.
37. Baker and Hargreaves (2001) Wind tunnel evaluation of a vehicle pollution dispersion model. *Journal of Wind Engineering and Industrial Aerodynamics* 89(2): 187-200.
38. Tominaga, Y., & Stathopoulos, T. (2013) CFD simulation of near-field pollutant dispersion in the urban environment: A review of current modeling techniques. *Atmospheric Environment* 79:716-730.
