# Supplementary material for: Methane emissions from natural gas vehicles in China
Source: Nat Commun. 2020 Sep 11;11:4588. doi: 10.1038/s41467-020-18141-0 (PMC7486943; doi:10.1038/s41467-020-18141-0)
Supplement: Supplementary file 2 — Reporting Summary [file 41467_2020_18141_MOESM2_ESM.pdf]

nature research

Corresponding author(s): Mark A. Zondlo
Da Pan

Last updated by author(s): Jun 30, 2020

Reporting Summary

Nature Research wishes to improve the reproducibility of the work that we publish. This form provides structure for consistency and transparency in reporting. For further information on Nature Research policies, see Authors & Referees and the Editorial Policy Checklist .

Statistics

For all statistical analyses, confirm that the following items are present in the figure legend, table legend, main text, or Methods section.

n/a	Confirmed	
☐	☒	The exact sample size (n) for each experimental group/condition, given as a discrete number and unit of measurement
☐	☒	A statement on whether measurements were taken from distinct samples or whether the same sample was measured repeatedly
☒	☐	The statistical test(s) used AND whether they are one- or two-sided<br>*Only common tests should be described solely by name; describe more complex techniques in the Methods section.*
☒	☐	A description of all covariates tested
☐	☒	A description of any assumptions or corrections, such as tests of normality and adjustment for multiple comparisons
☐	☒	A full description of the statistical parameters including central tendency (e.g. means) or other basic estimates (e.g. regression coefficient) AND variation (e.g. standard deviation) or associated estimates of uncertainty (e.g. confidence intervals)
☒	☐	For null hypothesis testing, the test statistic (e.g. F, t, r) with confidence intervals, effect sizes, degrees of freedom and P value noted<br>*Give P values as exact values whenever suitable.*
☒	☐	For Bayesian analysis, information on the choice of priors and Markov chain Monte Carlo settings
☒	☐	For hierarchical and complex designs, identification of the appropriate level for tests and full reporting of outcomes
☒	☐	Estimates of effect sizes (e.g. Cohen's d, Pearson's r), indicating how they were calculated

Our web collection on statistics for biologists contains articles on many of the points above.

Software and code

Policy information about availability of computer code

Data collection	10 Hz observations of methane and carbon dioxide concentrations were collected using commercial software from LI-COR Biosciences, (Li7700_win-1.0.18.exe and li7500rs_win-6.5.2.exe). 10 Hz observations of ammonia, carbon monoxide, and GPS location were collected using a custom LabVIEW code (LV12) .
Data analysis	Anaconda Python 2.7 distribution (Anaconda2-2019.10-Windows-x86_64) was used to analyze the data. The codes were provided with the supplementary data and were archived in https://github.com/dp7-PU/CH4_from_NGV_in_China.

For manuscripts utilizing custom algorithms or software that are central to the research but not yet described in published literature, software must be made available to editors/reviewers. We strongly encourage code deposition in a community repository (e.g. GitHub). See the Nature Research guidelines for submitting code & software for further information.

Data

Policy information about availability of data

All manuscripts must include a data availability statement. This statement should provide the following information, where applicable:

- Accession codes, unique identifiers, or web links for publicly available datasets
- A list of figures that have associated raw data
- A description of any restrictions on data availability

Time series of raw observations used for emission ratio calculations (10 Hz), time series of $\Delta CH_4:\Delta CO_2$, and $\Delta NH_3:\Delta CO_2$ and their determination coefficients (R2) are included in the Supplementary Data. The source code for calculating enhancement ratios and R2 is also provided with the supplementary data. Other data related to emission calculation are listed in the main text or in supplementary information.

Field-specific reporting

Please select the one below that is the best fit for your research. If you are not sure, read the appropriate sections before making your selection.

☐ Life sciences ☐ Behavioural & social sciences ☒ Ecological, evolutionary & environmental sciences

For a reference copy of the document with all sections, see nature.com/documents/nr-reporting-summary-flat.pdf

Ecological, evolutionary & environmental sciences study design

All studies must disclose on these points even when the disclosure is negative.

Study description	During the 2014 CAREBEIJING North China Plain field campaign, we deployed a mobile laboratory to quantify methane emissions from natural gas vehicles in China. Our mobile laboratory was equipped with fast response laser-based sensors to measure methane emissions from exhaust and leakage from natural gas buses and taxis in Baoding and Shijiazhuang.
Research sample	We captured emissions from 73 natural gas buses and 63 natural gas taxis during the field campaign. Out of the 73 natural gas buses, 39 of them were powered by liquefied natural gas and 34 were powered by compressed natural gas. The samples were randomly selected in the field to represent fleet level emissions in the two cities (~1000 for NG buses and ~2000 for NG taxis). The buses and taxis in the cities represent typical engine and emission control technologies in the country at the time of the field campaign (China Emission Standard V).
Sampling strategy	Vehicles were followed randomly to measure real-world emissions. The sample size was determined by counting the number of NGVs along this road and balanced by measurement constraints such as distance, drive time, battery capacities for the sensing package, and meteorological considerations. The sample size was so far the largest to our knowledge, not only for similar studies for NGVs in China but also for NGVs in other regions. Our results show small variations across the daily results and small differences between vehicles powered by compressed natural gas and liquefied natural gas, indicating the representativeness of our samples. We also surveyed engine types used by natural gas vehicles in China, which shows the sampled vehicles in the two cities represent the typical technology used in China (China V standard), justifying our extrapolation of our results to national level.
Data collection	Da Pan, Kang Sun, and Lei Tao drove the mobile lab on-road to measure emissions from vehicles.
Timing and spatial scale	We measured 26 hours on road, covering around 600 km in these two cities on June 10th, 11th, and 12th in 2014. Each measurements includes 10s to 200s measurement time, corresponds several hundred meters to several kilometers in spatial range. This spatial scale represents typical urban driving cycle.
Data exclusions	No data were excluded from data analysis.
Reproducibility	We sampled on different days and different locations to improve the representativeness of our observations. The time between measuring an individual NGV ranges from few tens of seconds to an hour depending on the traffic conditions. As mentioned before, the observed variation was very small.
Randomization	The vehicles were followed randomly on-road.
Blinding	Since the vehicles were followed randomly on-road, we considered our measurements blinded to the vehicle owners.

Did the study involve field work? ☒ Yes ☐ No

Field work, collection and transport

Field conditions	Ambient temperature was around 30 deg with no precipitation.
Location	The observations were made in Baoding (38°50'59.99" N 115°28'59.99" E) and Shijiazhuang (38°02'29.00" N 114°28'43.00" E).
Access and import/export	We did not import or export samples.
Disturbance	No disturbance was caused by our study.

Reporting for specific materials, systems and methods

We require information from authors about some types of materials, experimental systems and methods used in many studies. Here, indicate whether each material, system or method listed is relevant to your study. If you are not sure if a list item applies to your research, read the appropriate section before selecting a response.

Materials & experimental systems

n/a	Involved in the study	
☒	☐	Antibodies
☒	☐	Eukaryotic cell lines
☒	☐	Palaeontology
☒	☐	Animals and other organisms
☒	☐	Human research participants
☒	☐	Clinical data

Methods

n/a	Involved in the study	
☒	☐	ChIP-seq
☒	☐	Flow cytometry
☒	☐	MRI-based neuroimaging
