# Supplementary material for: Methane emissions from natural gas vehicles in China
Source: Nat Commun. 2020 Sep 11;11:4588. doi: 10.1038/s41467-020-18141-0 (PMC7486943; doi:10.1038/s41467-020-18141-0)
Supplement: Supplementary file 3 — Description of Additional Supplementary Files [file 41467_2020_18141_MOESM3_ESM.pdf]

Description of Additional Supplementary Files

File Name: Supplementary Data 1
Description: The dataset contains the 10 Hz observations and the python codes that were used to calculate methane emissions from natural gas vehicles.

File Name: Supplementary Movie 1
Description: The movie shows a video clip of our on-road measurements in 2014 (upper left panel) along with time series of CO_2 enhancement, CH_4 enhancement, slope and determination coefficient (R^2) of an orthogonal distance regression (ODR) of CO_2 enhancement and CH_4 enhancement within a time window of ±1 second (right panel). The data used for the ODR are presented in the lower left panel.
